# An NMF-based approach to discover overlooked differentially expressed gene regions from single-cell RNA-seq data

**DOI:** 10.1093/nargab/lqz020

**Published:** 2019-12-16

**Authors:** Hirotaka Matsumoto, Tetsutaro Hayashi, Haruka Ozaki, Koki Tsuyuzaki, Mana Umeda, Tsuyoshi Iida, Masaya Nakamura, Hideyuki Okano, Itoshi Nikaido

**Affiliations:** 1 Medical Image Analysis Team, RIKEN Center for Advanced Intelligence Project, Nihonbashi 1-chome Mitsui Building 15F, 1-4-1 Nihonbashi, Chuo-ku, Tokyo 103-0027, Japan; 2 Laboratory for Bioinformatics Research, RIKEN Center for Biosystems Dynamics Research, 2-1 Hirosawa, Wako, Saitama 351-0198, Japan; 3 Center for Artificial Intelligence Research, University of Tsukuba, 1-1-1 Tennodai, Tsukuba, Ibaraki 305-8577, Japan; 4 Bioinformatics Laboratory, Faculty of Medicine, University of Tsukuba, 1-1-1 Tennodai, Tsukuba, Ibaraki 305-8577, Japan; 5 Department of Orthopaedic Surgery, Keio University School of Medicine, 35 Sinanomachi, Shinjuku-ku, Tokyo 160-8582, Japan; 6 Department of Physiology, Keio University School of Medicine, 35 Sinanomachi, Shinjuku-ku, Tokyo 160-8582, Japan; 7 Bioinformatics Course, Master’s/Doctoral Program in Life Science Innovation (T-LSI), School of Integrative and Global Majors (SIGMA), University of Tsukuba, 2-1 Hirosawa, Wako, Saitama 351-0198, Japan

## Abstract

Single-cell RNA sequencing has enabled researchers to quantify the transcriptomes of individual cells, infer cell types and investigate differential expression among cell types, which will lead to a better understanding of the regulatory mechanisms of cell states. Transcript diversity caused by phenomena such as aberrant splicing events have been revealed, and differential expression of previously unannotated transcripts might be overlooked by annotation-based analyses. Accordingly, we have developed an approach to discover overlooked differentially expressed (DE) gene regions that complements annotation-based methods. Our algorithm decomposes mapped count data matrix for a gene region using non-negative matrix factorization, quantifies the differential expression level based on the decomposed matrix, and compares the differential expression level based on annotation-based approach to discover previously unannotated DE transcripts. We performed single-cell RNA sequencing for human neural stem cells and applied our algorithm to the dataset. We also applied our algorithm to two public single-cell RNA sequencing datasets correspond to mouse ES and primitive endoderm cells, and human preimplantation embryos. As a result, we discovered several intriguing DE transcripts, including a transcript related to the modulation of neural stem/progenitor cell differentiation.

## INTRODUCTION

The advancement of single-cell technology has enabled to investigate various tissues (1,2) and species (3,4) with single-cell RNA sequencing (scRNA-seq), which enables comprehensive cell typing and the elucidation of cell compositions and dynamics. In particular, scRNA-seq can reveal the subtle differences among cell states, such as intermediate stages of differentiation. By investigating differentially expressed (DE) genes among such cell states, we can elucidate regulatory processes including cell fate determination (5). In addition to traditional gene-level differential expression analyses, various novel analyses have been proposed for scRNA-seq studies, including the detection of differential distributions of expression levels (6) and differential splicing (7,8), isoform-level differential pattern analysis (9), discriminative learning approach for differential expression analysis (10) and dynamic prediction through the comparison of spliced and unspliced mRNAs (11). Thus, the development of various computational analysis methods that utilize information at the single-cell level is essential to advance the current understanding of RNA biology.

Recent comprehensive analyses of RNA-seq data have revealed the existence of various overlooked transcripts (12). For example, a comprehensive tumor analysis revealed that many tumors contain aberrant splicing patterns (neojunctions) that are not detected in normal samples (13). Additionally, numerous genetic variants are related to aberrant splicing associated with certain diseases (14). Therefore, it is important to detect novel splicing patterns, as well as detect differential expression of annotated transcripts. The transcriptomes of unstudied cell types, including rare cell types, can be revealed by scRNA-seq analyses, and we can now discover such cell type-specific splicing events.

In addition to major types of alternative splicing (AS), underappreciated classes of AS events, such as retained introns and microexons, are known to have essential roles, for example, in neuronal development (15). Intron retention, which is common in tumors, can generate peptides and be a source of neoepitopes for cancer vaccines, and therefore the detection of novel intron retention events is medically important (16). Furthermore, alternative polyadenylation, which produces isoforms that have 3′-untranslated regions (UTRs) of different lengths, is also known to be associated with several biological processes (17).

To reveal such complex AS patterns, several computational approaches have been developed that can detect previously unannotated splicing patterns. For example, spliced aligned reads (exon–exon junction reads) are beneficial in identifying the spliced mRNA structures (18,19). As another example, non-negative matrix factorization (NMF) has been used to decompose data into essential patterns and predict AS patterns from microarray data (20) and RNA-seq data (21).

In addition to these complex AS patterns, other types of transcripts, such as antisense transcripts transcribed from gene regions, are known to be essential regulators of gene expression (22). In light of such complex transcript structures, typical differential expression analysis based on previously annotated transcript structures might overlook some important DE genes (12). To find DE genes without relying on existing annotation, distinct approaches have been proposed that identify DE regions from read coverage data (23,24).

In single-cell RNA-seq technologies, there are two different trends: one aims to quantify the expression of huge number of cells to understand cellular composition and function, and the other aims to quantify the comprehensive gene expression of each cell to deepen our understanding of precise gene expression landscape. In this research, we used the data of latter scRNA-seq technologies to reveal complex transcript structures. Full-length scRNA-seq data such as Smart-seq (25,26) provide powerful data that can reveal these structures. Other scRNA-seq protocols, such as SUPeR-Seq (27), which can capture non-poly(A) transcripts, will also be useful to detect various overlooked DE transcripts. In particular, we have developed a single-cell full-length total RNA-seq (RamDA-seq) method and have validated that it precisely captures full-length transcripts and also captures various types of RNAs such as enhancer RNAs (28). By utilizing such scRNA-seq data, we can perform differential expression analyses between cell states more precisely.

**Figure 1.**
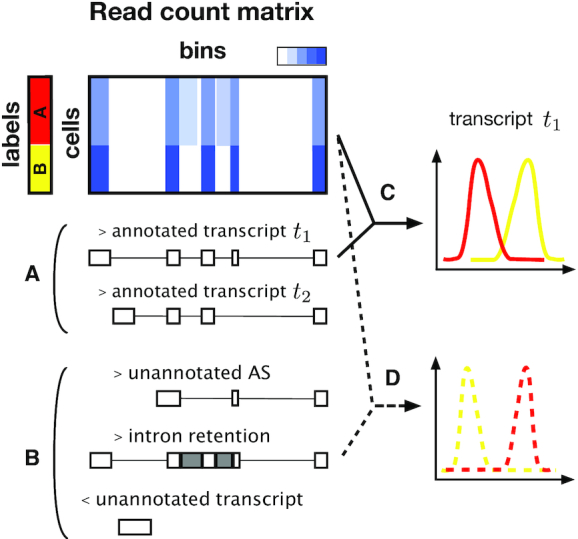
Graphical abstract of the overlooked ODEGR. Coverage of scRNA-seq data and annotated transcripts in the region (**A**) and previously unannotated transcripts such as novel alternative splicing patterns, intron retention and unannotated antisense transcripts (**B**). Although annotation-based expression profiling and the following differential expression analysis is an effective approach to find DE transcripts (**C**), such a method might overlook the differential expression of unannotated transcripts (**D**).

**Figure 2.**
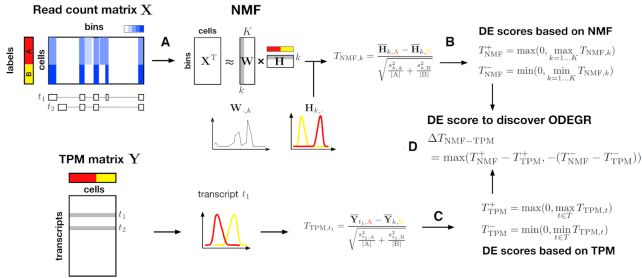
Graphical abstract of our algorithm to discover ODEGRs. First, we use NMF to decompose the mapped read count matrix ($\mathbf {X}$) for a gene region (**A**), and then use *t*-statistics to quantify the differential expression level while keeping the positive maximum and negative minimum values (**B**). We also quantify the differential expression level using an annotation-based expression profile (in a transcripts per million (TPM) matrix) (**C**). At last, we quantify the unexpectedness of differential expression based on the above values (**D**).

Accordingly, we have developed an approach to discover **O**verlooked **D**ifferentially **E**xpressed **G**ene **R**egions (ODEGRs), which is derived from several kinds of transcripts such as novel AS patterns, intron retention and antisense transcripts, to complement the annotation-based differential expression analysis of single-cell data (Figure 1). Our approach utilizes the composition of scRNA-seq data, which contain information from many samples (i.e. cells), and decomposes the mapped count data for gene regions using NMF. With NMF, we can computationally extract reproducible signals corresponding to transcript structures and their associated expression profiles without relying on transcript annotations (Figure 2A). In addition, the non-negative constraint of NMF, which is its principal difference from other matrix decomposition methods, is effective in preserving the relation of the magnitude of expression. Next, we developed the following scores for a gene region: $T_{\text{NMF}}^{\pm }$, $T_{\text{TPM}}^{\pm }$, and Δ*T*_NMF − TPM_. $T_{\text{NMF}}^{\pm }$ represents the scores that quantify the differential expression levels between two groups based on the NMF result (Figure 2B), while $T_{\text{TPM}}^{\pm }$ represents the scores that quantify the differential expression levels for annotation-based expression data (Figure 2C). Thus, Δ*T*_NMF − TPM_ represents the score that quantifies the differential expression that is not detected in the annotation-based approach (Figure 2D). We investigated gene regions with high Δ*T*_NMF − TPM_ values in order to discover ODEGRs.

We applied our algorithm to three real datasets: (i) mouse embryonic stem (ES) cells and primitive endoderm (PrE) cells, (ii) neural stem cells (NSCs) derived from human induced pluripotent stem (iPS) cells and (iii) human day 3 and day 4 embryonic cells. First, we evaluated whether the NMF-based approach could quantify and find local DE regions from simulated data. We also evaluated whether it could detect AS switches within a gene, as determined by annotation-based analysis. Our algorithm was indeed able to detect such DE regions without relying on transcript annotations. Then, we applied our method to real datasets to detect ODEGRs and found several intriguing examples. From the perspective of previous research, our results correspond, for example, to unannotated splicing patterns, antisense transcript and unannotated 3′-UTRs of adjacent genes. In particular, some ODEGRs are related to critical regulatory mechanisms such as the modulation of differentiation and tissue-specific imprinting. Thus, our novel differential expression analysis method identified some important ODEGRs and can complement annotation-based methods, making it a useful method for analysis in the increasing number of scRNA-seq experiments.

## MATERIALS AND METHODS

### NMF-based approach for discovering ODEGR

In this research, we focused on detecting DE gene regions that were overlooked in the differential expression analysis of previously annotated transcripts from mapped read count data. We divided a gene region into 100-bp bins and described a read count matrix for a gene region with a *C* × *L* matrix $\mathbf {X}$, where *C* is the number of cells and *L* is the number of bins (The effect of bin size is shown in Supplementary Paragraph 2, and our method is robust for bin size). We investigated the property of $\mathbf {X}$ and showed that full-length scRNA-seq datasets do not contain many missing values (see Supplementary Paragraph 3). First, we decomposed $\mathbf {X}$ into two non-negative matrices (using NMF):

(1) $$\begin{eqnarray*} \mathbf {X}^{\mathrm{T}} \approx \mathbf {W}\mathbf {H} \end{eqnarray*}$$

where $\mathbf {W}$ and $\mathbf {H}$ are *L* × *K* and *K* × *C* non-negative matrices (*K* is the factorization rank) referred to as ‘metagenes’ and ‘metagene expression profiles’ in previous studies, respectively (29,30). In this research, we hypothesized that $\mathbf {W}$ corresponds to the transcript structure including splicing patterns and that $\mathbf {H}$ corresponds to the expression for each structure in each cell.

Second, we quantified the differential expression level of a structure *k* ∈ (1...*K*) between two groups *A* and *B* based on Welch’s *t*-test:

(2) $$\begin{eqnarray*} T^{(K)}_{{\text{NMF}} ,k} = \frac{\overline{\mathbf {H}}_{k,C_A} - \overline{\mathbf {H}}_{k,C_B} }{\sqrt{\frac{s_{k,A}^2}{|C_A|}+\frac{s_{k,B}^2}{|C_B|}}}, \end{eqnarray*}$$

where *C*_*A*_ is the list of cells whose labels are *A* and $\overline{\mathbf {H}}_{k,C_A}$, $s_{k,A}^2$, and |*C*_*A*_| are the sample mean of $\mathbf {H}_{k,\cdot }$, variance, and size of group *A*, respectively. Owing to the non-negative constraint, the relation between the two groups (i.e. $\overline{\mathbf {H}}_{k,C_A} - \overline{\mathbf {H}}_{k,C_B}$ can be greater or smaller than 0) will be consistent with the relation in the original expression space. Our goal was to identify overlooked differential expression, and therefore, such relations, as well as their absolute values, were effective indicators for discovering ODEGRs. Therefore, we defined the following two scores, which correspond to the relation $\overline{\mathbf {H}}_{k,C_A} >\overline{\mathbf {H}}_{k,C_B}$ and $\overline{\mathbf {H}}_{k,C_A} < \overline{\mathbf {H}}_{k,C_B}$, respectively:(3)
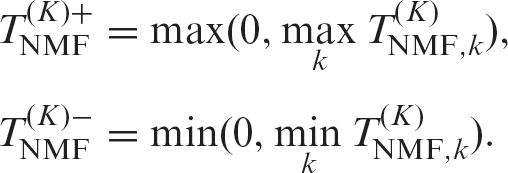


In NMF, the factorization rank (*K*) must be decided in advance, and the value is critical for analytical results. The various transcript structures cannot be separated with small *K* values and are excessively separated with large *K* values. In either case, the expression profiles become ambiguous, and we might overlook the DE regions if an inappropriate *K* value is selected. Therefore, we decomposed the data with several *K* values (*K* ∈ (2, 5, 10) in this research) and calculated the positive maximum and negative minimum values:(4)
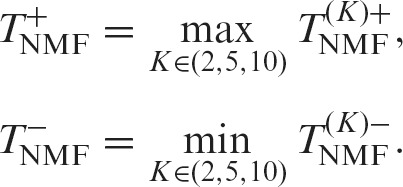


Next, we defined similar scores for the TPM (transcripts per million) matrix, which represents the expression profile based on annotated transcripts (we used log_10_(TPM + 1) in actuality). We described the list of transcripts for the gene region using *T* and calculated Welch’s *t*-statistic as before for a transcript *t* ∈ *T*, which is referred to as *T*_TPM, *t*_. Then, the scores for the gene region were defined by the positive maximum and negative minimum among transcripts of the gene as follows:(5)
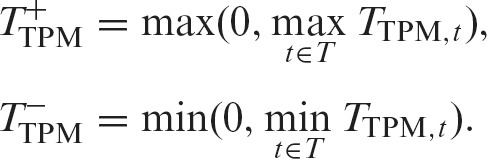


At last, we developed a score to detect ODEGRs as follows:(6)
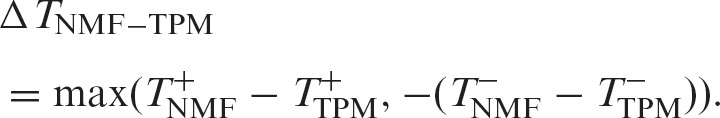
Because these is no global NMF optimization algorithm, we calculated Δ*T*_NMF − TPM_ using three different random seeds and also used minimum Δ*T*_NMF − TPM_ to obtain reliable ODEGRs. We investigated the ODEGRs based on their ranked Δ*T*_NMF − TPM_ values in descending order, and detailed procedure, pseudo-code, and the reproducibility of NMF results are described in the Supplementary Paragraph 9.

We also developed a score Δ*T*_NMF − Mean_ that measured the overlooked differential expression merely using the mean of the coverage. We used this score to evaluate whether the NMF-based approach separates the signal and detects complex DE patterns. We calculated the mean of the logarithm of data for a cell *c*($\left(\sum\nolimits_{l=1}^L \log _{10}(\mathbf {X}_{c,l}+1)/L\right)$, where *L* is the number of bins) as well as the corresponding Welch’s *t*-statistic as before and Δ*T*_NMF − Mean_ likewise.

### Dataset

In this research, we used scRNA-seq data from the following three experiments.

#### mES-PrE dataset

The first dataset is derived from mouse ES cells and primitive endoderm (PrE) cells subjected to RamDA-seq and was examined in our previous study (28). We used the data from 5G6GR mouse ES cells samples at 0 and 72 h after dexamethasone induction and defined the cell type at each time point as ES cells (92 cells) and PrE cells (93 cells), respectively.

#### hNSC-NC dataset

The second dataset corresponds to human NSCs derived from iPS cells measured by RamDA-seq. There is heterogeneity within the population, and some subpopulations other than the NSC subpopulation were identified (Supplementary Paragraph 1 and Figure S1). After clustering these cells and defining the cell types based on marker gene expression, we identified 515 NSCs and 80 partially differentiated neural cells (NCs).

#### hE3-E4 dataset

The third dataset is measured by different scRNA-seq technology, Smart-seq2 (26), and is derived from Smart-seq2 for human preimplantation embryos (31). We used the data from day 3 embryonic (E3) cells (81 cells) and day 4 embryonic (E4) cells (190 cells).

### Data processing

The mouse ES-PrE dataset was derived from our previous work (28), and we regarded cells 0 h and 72 h after induction as ES and PrE cells, respectively. The scRNA-seq reads were aligned to the mouse mm10 genome using HISAT2(v2.0.1) (32) with the parameters ‘–dta-cufflinks -p 4 -k 5 -X 800 –sp 1000,1000,’ and uniquely mapped reads were selected using the BAMtools ‘filter’ command with the parameters ‘-isMapped true -tag NH:1’ and the SAMTools ‘view’ command with the parameter ‘-q 40.’ The genome-wide coverage data were generated from these mapped data using the ‘bamCoverage’ command in deepTools(2.7.10) (33) with the parameters ‘–binSize 1 –smoothLength 1 –normalizeUsingRPKM.’ We also quantified transcript-level expression data (i.e. TPM matrix) from scRNA-seq data using the Sailfish(v0.9.2) (34) ‘quant’ command with the parameter ‘-l U’ and GENCODE vM9 annotation.

The human NSC-NC dataset was measured using RamDA-seq for cell populations derived from NSCs differentiated from iPS cells. The scRNA-seq reads were aligned to the human hg38 genome with STAR(v2.5.2a) (35), and the coverage data was constructed with ‘bamCoverage’ command as mentioned above. We also quantified the transcript-level expression data (TPM matrix) with Sailfish(v0.10.0) based on GENCODE v24 gene annotation. Based on the known marker gene expression, we identified subpopulations in the data (see Supplementary Figure S1). In particular, we found that a subpopulation expressed some stemness marker genes, such as *SOX2*, *LIN28* and *POU5F1*, and another subpopulation expressed neural marker genes, such as *ASCL1*. We regarded the cell types corresponding to those two subpopulations as NSCs and NCs, respectively.

The human E3–E4 dataset was derived from Smart-seq2 dataset produced by Petropoulos et al. (31). The scRNA-seq reads were aligned to the human hg38 genome with STAR, and the coverage data was constructed with ‘bamCoverage’ command as mentioned above, and transcript-level expression data (TPM matrix) was calculated with Sailfish based on GENCODE v24 gene annotation. We also calculated the TPM matrix with Salmon(v0.14.1) (36) and confirmed that Δ*T*_NMF − TPM_ with Sailfish and that with Salmon were almost equivalent (see Supplementary Paragraph 7). Due to the data processing failure, we removed one E3 cell data and used 80 E3 cells and 190 E4 cells for our algorithm.

For all datasets, we generated a mapping count data matrix for each gene region as follows. First, we extracted the transcript list so that the mean expression of a transcript *t* is over a set threshold (i.e. ∑_*c*_log_10_(TPM_*t*, *c*_ + 1)/*C* > 0.5, where *C* is the number of cells). Next, we constructed the unique protein-coding gene list, which corresponds to the above transcript list. Then, we selected 6921, 9359 and 5486 genes from each dataset and constructed a count data matrix (100-bp bins) for each gene region from the genome-wide coverage data of each cells. The gene regions were defined by the genomic start location and end location of the row of the gene in the GENCODE GTF files (vM9 for the mES-PrE dataset and v24 for the hNSC-NC dataset and hE3-E4 dataset). We filtered the bins that contained various genes because the target gene might falsely be regarded as occurring in an ODEGR owing to the differential expression of other overlapping genes. We also filtered the bins that were derived from regions with low mappability. This is because such bins might falsely be regarded as a DE region owing to the misalignment of reads. In this research, we defined bins with low mappability as those for which the minimum of 24-bp mappability (downloaded from https://bismap.hoffmanlab.org (37)) was 0.5 or less. Then, the genes that remained with bin sizes under 100 were filtered. In this way, 4965, 6491 and 2230 genes were selected for differential expression analysis.

### Implementation and computational cost

We computed NMF with the *NMF* package in the R statistical computing environment (30) and used the objective function based on the Euclidean distance between the data matrix $\mathbf {X}$ and the reconstructed matrix $\mathbf {W}\mathbf {H}$ as calculated by factorization (38). We also implemented our algorithm that used sparse NMF (39), and results are shown in Supplementary Paragraph 6. The raw count matrix data has excessively large values in some bins, and such large values cause the underestimation of the influence of the remaining bins in the objective function. Therefore, we applied a log_10_(count + 1) transformation to the count values before NMF calculation. The scripts are available at GitHub (https://github.com/hmatsu1226/ODEGRfinder).

Since the NMF calculations of all gene regions are independent from each other, we performed NMF for each gene region in parallel using Sun Grid Engine. In the NMF analysis with *K* = 10 for the first 1000 gene regions, the computational times were about 1.7 and 10.9 h with maximum memory usage of about 240 and 544 Mb for the mES-PrE and hNSC-NC datasets, respectively.

### Validation method

#### Simulation dataset

We constructed simulation data from the mES-PrE dataset. First, we calculated the mean of the logarithm of the coverage of a gene region $\left(\sum _{l=1}^L \log _{10}(\mathbf {X}_{c,l}+1)/L\right)$, where *L* is the number of bins and *c* is the index of a cell. We then calculated the *p*-value of the *t*-test comparing this value between the ES cells and PrE cells and extracted the top 100 most significant DE genes. Second, we randomly selected a sample of count data ($\mathbf {X}$) from these 100 DE genes, and reshaped the *C* × *L* matrix $\mathbf {X}$ into a *C* × *L*′ matrix $\mathbf {X}^{\prime }$ (*L*′ < *L*) by averaging $\mathbf {X}_{c,i}$ from *i* = ⌊(*b* − 1)(*L* − 1)/*L*′⌋ to ⌊*b*(*L* − 1)/*L*′⌋ for each bin *b* corresponding to $\mathbf {X}^{\prime }_{c,b}$. Then, we randomly selected a gene from among 4,965 genes and combined the count data for the gene using the above matrix $\mathbf {X}^{\prime }$ so that the combined matrix had the local DE pattern. However, if the two selected genes had the same DE trend, that is, both satisfied −log_10_(*p*-value) > 10 for the same side in the corresponding *t*-test, the combined matrix did not have the local DE pattern, and so we selected one of the 4965 genes at random again. We generated a positive-control datasets with 1000 datapoints as above for *L*′ = 10, 50 and 100, and we regarded the raw data as the negative-control set.

#### Alternative isoform expression definition

We defined genes with alternative isoform expression based on the TPM matrix. We defined a gene that satisfied −log_10_(*p*-value) for a corresponding *t*-test for $T^{+}_\text{TPM}$ and $T^{-}_\text{TPM}$ over α as belonging to the positive-control set, and the remaining genes as belonging to the negative-control set. We used *α* = 5, 10 and 15 and the number of genes in the positive-control set were 75, 25 and 8 for the mES-PrE dataset and 333, 95 and 51 for the hNSC-NC dataset, respectively.

## RESULTS

### Validation on simulation dataset

At first, we investigated the performance of NMF-based differential expression quantification and whether our approach can quantify the local differences in a region using simulation data. We evaluated whether $T^{+}_\text{NMF}$ and $T^{-}_\text{NMF}$ are reasonable values to quantify the local differences. We compared these values to the *t*-statistics based on the difference of the mean read count in the local DE region and showed that these values are almost equivalent (see Supplementary Paragraph 4). We also calculated $T^{+}_\text{NMF}$ and $T^{-}_\text{NMF}$ for label-shuffled data and confirmed that these values did not become large by chance due to over-decomposing the read count matrix (see Supplementary Paragraph 4).

Next, we regarded the simulation and raw data as positive-control and negative-control datasets, respectively, and evaluated the ability to detect local DE regions based on Δ*T*_NMF − Mean_. We also compared the performance when we used *K* = (2, 5, 10), as mentioned in Equation (4), or one fixed value (i.e. *K* = 2, 5 or 10) for calculating $T^{+}_\text{NMF}$ and $T^{-}_\text{NMF}$.

**Figure 3.**
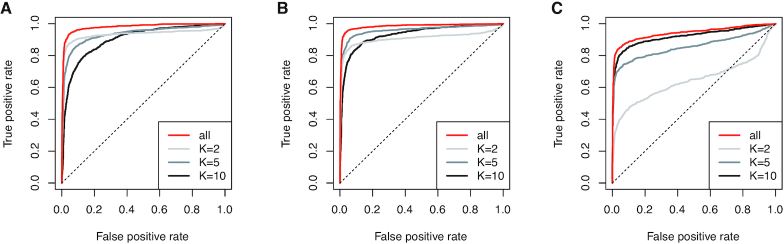
The ROC curves for the simulated dataset. Simulation results for (**A**) *L*′ = 100, (**B**) 50 and (**C**) 10, where *L*′ is the length of local differential expression patterns.

The area under the ROC curve (AUROC) values for *all*, *K* = 2, *K* = 5 and *K* = 10 were 0.98, 0.93, 0.93 and 0.90, respectively for simulation data with *L*′ = 100 (Figure 3A). The AUROC values for the *L*′ = 50 dataset were 0.98, 0.91, 0.96 and 0.93, respectively, and those for the *L*′ = 10 dataset were 0.94, 0.64, 0.86 and 0.94, respectively (Figure 3B and C). In all cases, our algorithm using multiple *K* values showed high performance, and therefore, our NMF-based approach is useful for discovering various local differences.

### Validation with alternative isoform expression

We also investigated whether the NMF-based approach can quantify the complex DE patterns associated with genes that have alternative isoform expression. Based on the TPM matrix calculated from the annotation, we defined the positive-control and negative-control datasets. The former consists of the gene set with different isoforms expressed in different groups, while the latter consists of the remaining genes (see the ‘Materials and Methods’ section for detailed definitions). Then, we evaluated the ability to detect such complex DE patterns based on Δ*T*_NMF − Mean_.

**Figure 4.**
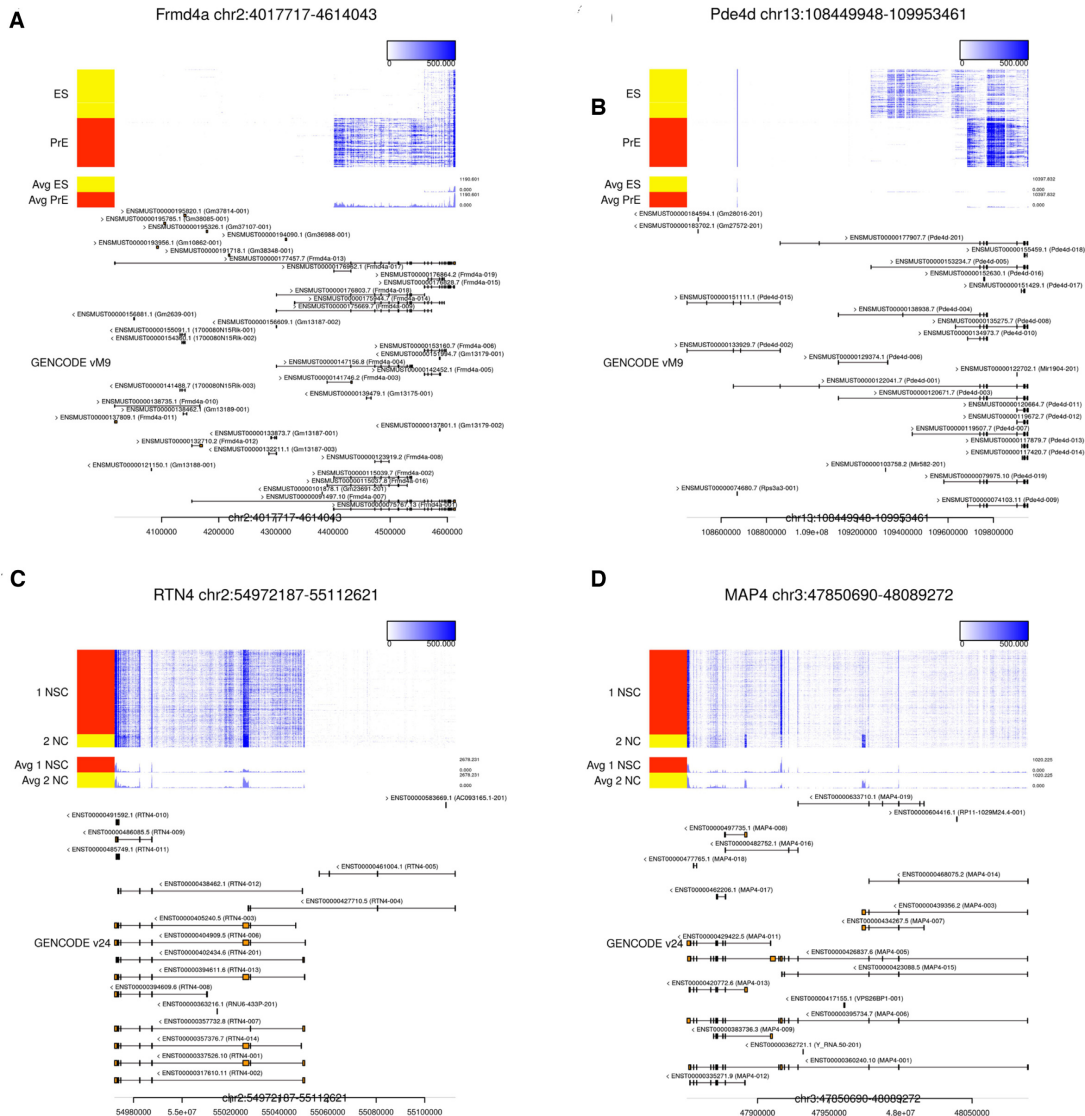
Examples of alternative isoform expression. The visualizations of read coverage and transcript annotations for (**A**) *Frmd4a*, (**B**) *Pde4d*, (**C**) *RTN4* and (**D**) *MAP4*, respectively. (A) and (B) are the examples from the mES-PrE dataset, while (C) and (D) are the examples from the hNSC-NC dataset. These figures are visualized with Millefy, which provides genome-browser-like visualizations of scRNA-seq datasets https://github.com/yuifu/millefy (51).

The positive-control examples of alternative isoform expression in the mES-PrE dataset were *Frmd4a* and *Pde4d*, which are known for frequent transcription start site (TSS) switching events (40) (Figure 4A and B). Based on our criteria, both *Frmd4a* and *Pde4d* were highly ranked (53rd and 23rd out of 4965 genes, respectively).

The examples in the hNSC-NC dataset were *RTN4*, also known as *NOGO*, which encodes the Nogo-A isoform that contains exon 3 and is expressed in neural precursor cells (41) (Figure 4C), and *MAP4*, which is known for its alternative isoform expression across neural cell types (42) (Figure 4D). These genes were highly ranked in our criteria (40th and 1st out of 6491 genes, respectively.) Thus, the typical genes with alternative isoform expression are highly ranked in our criteria Δ*T*_NMF − Mean_.

**Figure 5.**
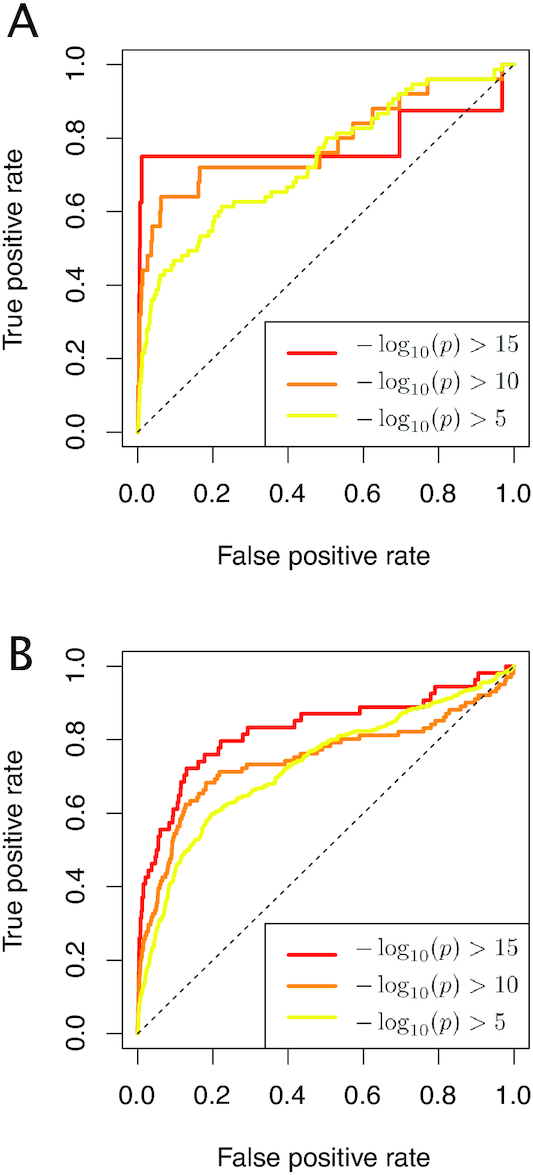
The ROC curves for detecting genes with alternative isoform expression. The results for the (**A**) mES-PrE and (**B**) hNSC-NC datasets.

Overall, the AUROC values (for threshold 15) were about 0.79 and 0.83 for the mES-PrE and hNSC-NC datasets, respectively (Figure 5). Although our algorithm overlooked some alternative expression patterns, the high AUCROC values demonstrated the effectiveness of our algorithm for discovering previously unannotated DE transcripts.

### Discovery of ODEGRs

Next, we investigated the existence of ODEGRs by using Δ*T*_NMF − TPM_. In brief, the values of Welch’s *t*-statistics based on NMF ($T^{+}_\text{NMF}$ and $T^{-}_\text{NMF}$) and TPM ($T^{+}_\text{TPM}$ and $T^{-}_\text{TPM}$) were highly correlated (Pearson’s correlation coefficients for the mES-PrE dataset, hNSC-NC dataset and hE3-E4 dataset were about 0.83, 0.84 and 0.77, respectively), and large Δ*T*_NMF − TPM_ values were observed for only a small fraction of genes (Supplementary Figure S6). Therefore, we ranked genes by Δ*T*_NMF − TPM_ in descending order to identify ODEGRs. Only a small fraction of genes had large positive values of Δ*T*_NMF − TPM_ (Supplementary Figure S6). Five genes in the mES-PrE dataset had Δ*T*_NMF − TPM_*Z*-scores over 3, 39 genes in the hNSC-NC dataset did, and 16 genes in the E3-E4 dataset did. Although the number of ODEGRs discovered by our algorithm were few, several intriguing ODEGRs were identified. We also proposed some approaches, such as permutation-based test, to evaluate the significance of these ODEGRs (see Supplementary Paragraph 8).

#### mES-PrE dataset

**Figure 6.**
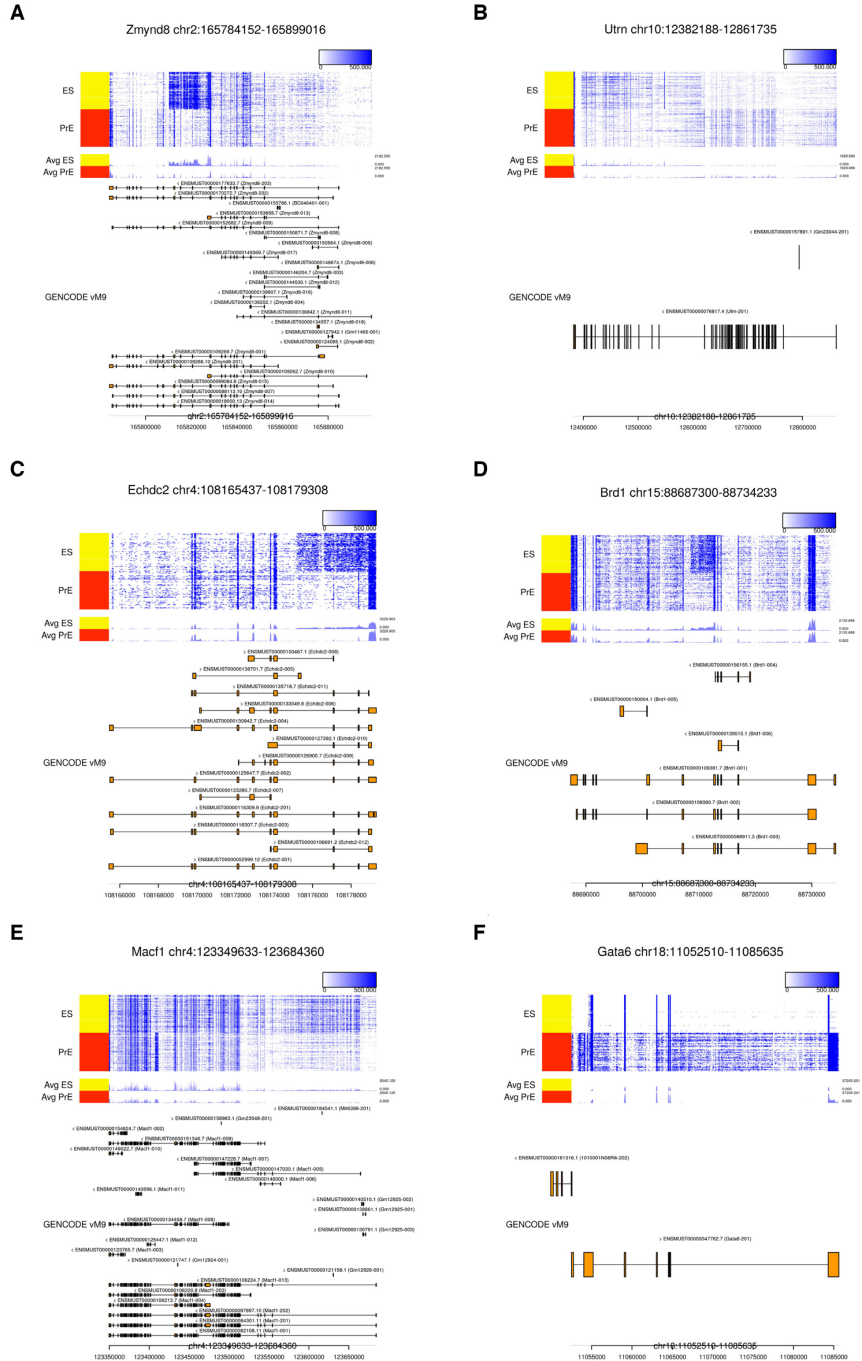
Examples of high-ranking genes in the mES-PrE dataset. The results for the six top-ranked genes (in descending order) (**A**) *Zmynd8*, (**B**) *Utrn*, (**C**) *Echdc2*, (**D**) *Brd1*, (**E**) *Macf1* and (**F**) *Gata6* are visualized.

The read coverage and transcript annotation for the six highest-ranking genes in the mES-PrE dataset are shown in Figure 6.

The 1st and 4th ranked genes were *Zmynd8* and *Brd1*, and numerous reads were mapped to the specific intron regions of these genes (Figure 6A and D). We confirmed the ES cell-specific expression of unannotated transcripts in these regions using qRT-PCR (see Supplementary Paragraph 9.5). The novel enhancer-associated antisense transcripts for these genes have previously been reported in mESCs (43), and this suggests that our approach can detect several kinds of DE transcripts, including antisense transcripts.

The 2nd ranked gene was *Utrn*, and two distinct coverage patterns of peaks that correspond to exons were observed in ES and PrE cells, respectively (Figure 6B). Since the annotation contains only one isoform, this DE pattern was overlooked in the annotation-based approach. We used GENCODE vM9 such that the analytical results were consistent with previous work (28), and we also considered the possibility that the latest annotation includes the isoforms corresponding to such patterns. We recalculated the TPM values using GENCODE vM18, and $T_\text{TPM}^{+}=46.2$ and $T_\text{TPM}^{-}=-15.5$ for vM18, in comparison to $T_\text{TPM}^{+}=0.0$ and $T_\text{TPM}^{-}=-4.4$ for vM9 (Supplementary Paragraph 9.2 and Figure S9). This result suggests the existence of DE transcripts that were not annotated in vM9. A similar result was observed for the 7th ranked gene *Arid5b* (Supplementary Figure S9). These results demonstrate the potential of our approach for discovering previously unannotated isoforms.

The 3rd ranked gene was *Echdc2*, which had numerous reads mapped to its 3′ intron region (Figure 6C). Although such a pattern is consistent with intron retention, this mapping pattern is continued from adjacent gene *Zyg11a* (see Supplementary Paragraph 9.3 and Figure S10). We performed qRT-PCR with several primer sets and confirmed ES cell-specific expression of the unannotated transcript (see Supplementary Paragraph 9.5). *Zyg11a* also shows ES cell-specific expression, and the reads at the 3 intron of *Echdc2* might correspond to an unannotated long isoform of *Zyg11a* that overlaps with the *Echdc2* region.

The 5th ranked gene was *Macf1*, and numerous reads were mapped to the specific intron region of the gene in PrE cells (Figure 6E). An exon was annotated for the region in vM18, and the DE transcript including the exon was overlooked in differential expression analysis using vM9, which was also the case for *Utrn* and *Arid5b* (Supplementary Figure S9).

The sixth ranked gene was *Gata6* (Figure 6F). The exogenous *Gata6*, which lacks a 3′-UTR end, is arbitrarily expressed in these ES cells. After dexamethasone induction, Gata6 is transported into the nucleus, ES cells differentiate into PrE cells, and the level of expressed endogenous *Gata6* increases. Because the annotation file does not include exogenous structure, annotation-based TPM cannot reflect the exogenous expression patterns, which resulted in high Δ*T*_NMF − TPM_ values.

#### hNSC-NC dataset

**Figure 7.**
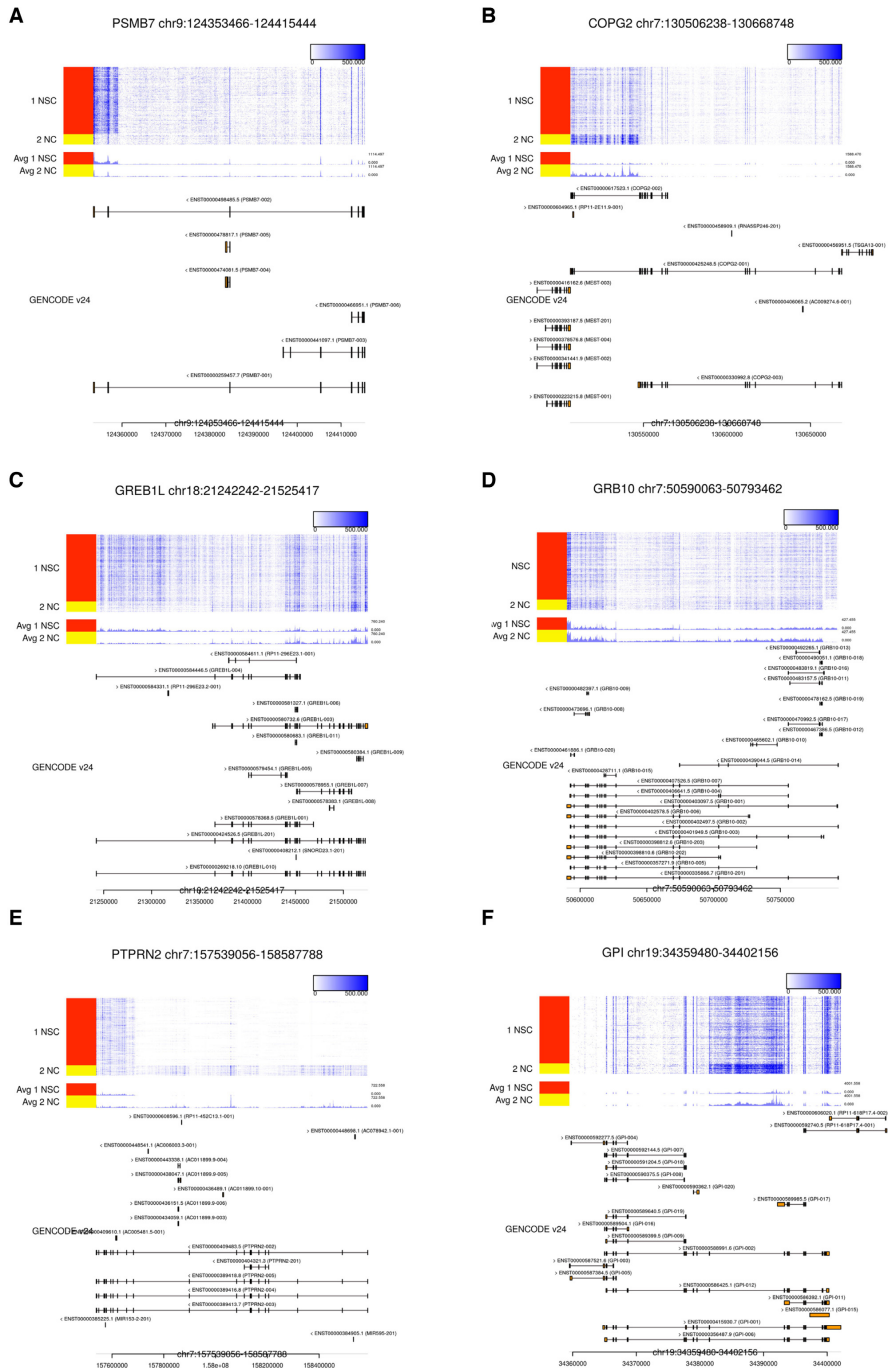
Examples of high-ranking genes in the hNSC-NC dataset. The results for (**A**) *PSMB7*, (**B**) *COPG2*, (**C**) *GREB1L*, (**D**) *GRB10*, (**E**) *PTPRN2* and (**F**) *GPI*, the 2nd, 6th, 10th, 15th, 17th and 18th ranked genes, respectively, are visualized.

In comparison to the results of the mES-PrE dataset, the results of the hNSC-NC dataset contained uninteresting patterns among the most highly ranked genes (Supplementary Paragraph 9.1 and Figure S8). Therefore, we show six high-ranking genes of great interest in the hNSC-NC dataset (Figure 7).

The second ranked gene was *PSMB7*, and many reads from NSCs were mapped to its 3′ intron region, which is similar to the result for *Echdc2* in the mES-PrE dataset (Figure 7A). The coverage pattern was continued from the adjacent gene *NEK6*, and the coverage of the intron region is correlated with the that of *NEK6* (Supplementary Figure S10). This result suggests the existence of an unannotated long transcript of *NEK6* that overlaps with the *PSMB7* region.

The sixth ranked gene was *COPG2*, and numerous reads were mapped to its 3′ intron regions, resembling the results for *Echdc2* and *PSMB7* (Figure 7B). These reads are also likely to be derived from transcripts of the adjacent gene *MEST*, which may have an unannotated long transcript. Intriguingly, in mouse, *Mest* is an imprinted gene, and a long isoform of *Mest* (referred to as *MestXL*) is expressed in the developing central nervous system, which results in the repression of *Copg2* on the same paternal allele (44). Therefore, the long transcript of *MEST* and the tissue-specific imprinting of *COPG2* depending on the long transcript are thought to occur in human. Thus, the detection of overlapping unannotated transcripts can be associated with regulatory mechanisms.

The 10th and 15th ranked genes were *GREB1L* and *GRB10*, and distinct AS patterns are suggested by the difference in mapped read counts between NSCs and NCs, especially for the intron region (Figure 7C and D). In *GREB1L*, several reads mapped to the 5′ intron region (left side of the heatmap in Figure 7C), and the long isoform appears to be expressed in NSCs. Our NMF-based algorithm detected such overlooked differences ($T_{\text{NMF}}^+=13.8$) in contrast to the annotation-based approach ($T_{\text{TPM}}^+=0.8$). Since RamDA-seq detects not only mature mRNAs but also pre-mRNAs, many reads mapped to intron regions are considered to be derived from pre-mRNA expression (28). Because the annotation-based algorithm does not usually use intron-mapped reads, our proposed algorithm that utilizes such information is effective for AS pattern identification, especially for genes with alternative TSSs.

For *GRB10*, numerous reads were mapped to its 5′ intron, and cell-type-specific TSS switching likely occurs for this gene (Figure 7D). *GRB10* is an imprinted gene and is known for its unique TSS switch mechanism in mouse (45). In the differentiation of mESCs into motor neurons, the expression of *Grb10* changes from the maternal to paternal allele. The upstream promoter is used for maternal expression, and the downstream alternative promoter is used for paternal expression. Therefore, the 5′ intron-mapped reads, which are detected in only NSCs, support the alternative TSS based on the above mechanism and reflect DE patterns, observable by utilizing intron reads.

The 17th ranked gene was *PTPRN2*, and long transcript is highly expressed in NCs, and besides, there appears to be a short unannotated transcript in NSCs (Figure 7E). Notably, in mouse, an alternative promoter exists downstream of *Ptprn2*, and the transcription from the promoter drives the miR-153 precursor transcript embedded in the *Ptprn2* gene region (46). Moreover, miR-153 is highly expressed in mouse neural stem/progenitor cells (NSPCs), and the repression of miR-153 leads to differentiation, and hence, miR-153 modulates NSPCs (47). Human miR-153 is located in *PTPRN2* (48), and therefore, the short transcript in the 3′ region is likely a key factor that distinguishes human NSCs and NCs but is overlooked by annotation-based analysis.

The 18th ranked gene was *GPI*, and numerous reads from NCs were mapped to its central intron region (Figure 7F). In *GPI*, the existence and conservation of a minisatellite in its intron have been reported (49). Although the increase in such reads might be an artifact caused by repetitive sequences, a NC-specific transcript might exist in the region.

#### hE3-E4 dataset

**Figure 8.**
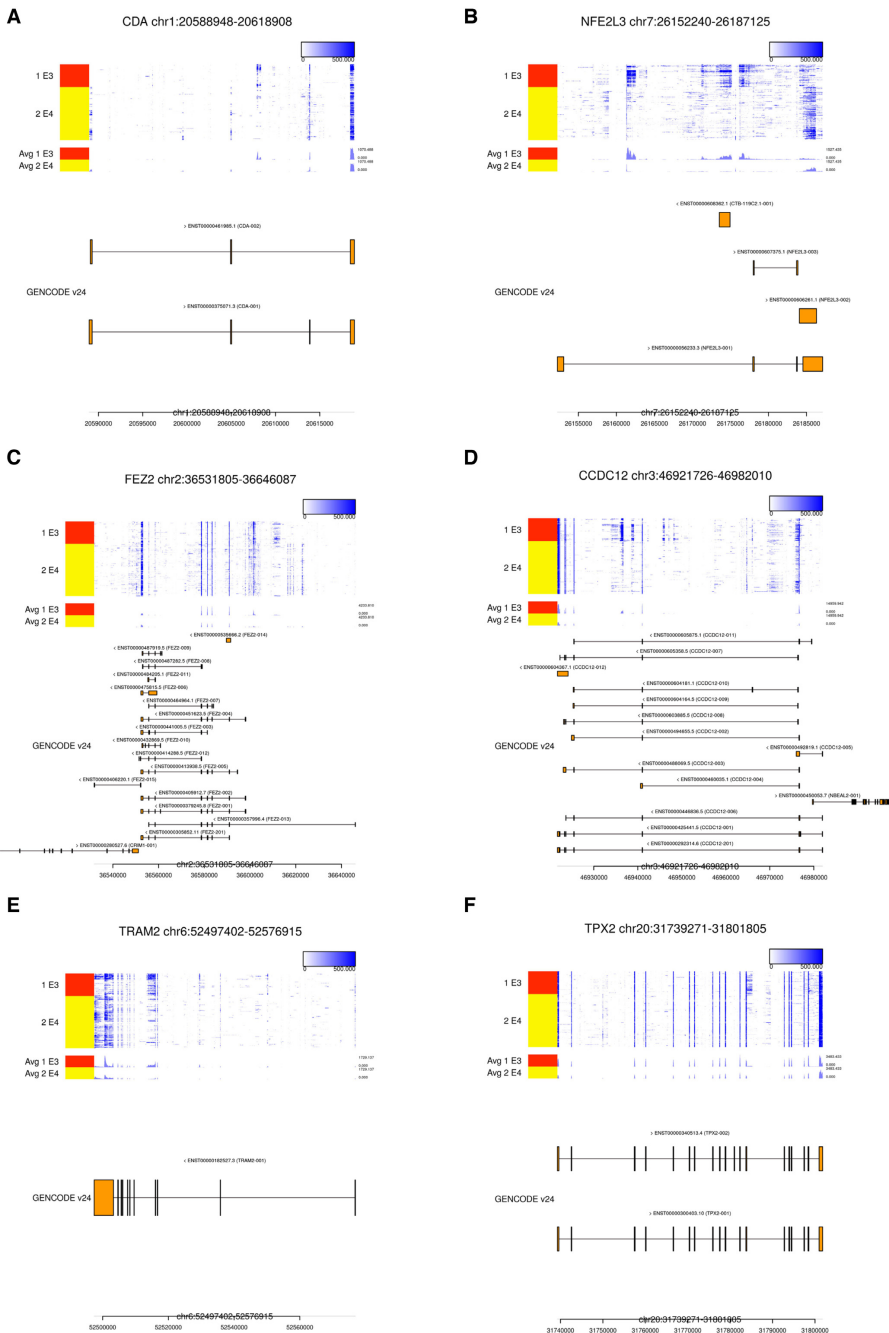
Examples of high-ranking genes in the hE3-E4 dataset. The results for (**A**) *CDA*, (**B**) *NFE2L3*, (**C**) *FEZ2*, (**D**) *CCDC12*, (**E**) *TRAM2* and (**F**) *TPX2*, the 3rd, 5th, 6th, 7th, 8th and 16th ranked genes, respectively, are visualized.

Unlike the previous two datasets that are derived from single-cell full-length total RNA-seq (RamDA-seq), the hE3-E4 dataset is derived from single-cell full-length polyA RNA-seq (Smart-seq2). We discovered several novel isoforms (Figure 8), and therefore, our algorithm will be useful for analyzing various single-cell full-length RNA-seq technologies.

The third ranked gene was *CDA* and numerous reads from E3 cells were mapped to specific intron region (Figure 8A). There were several spliced aligned reads between the region and CDA exon (Supplementary Figure S11), which suggest the existence of unannotated exon. The seventh ranked gene *CCDC12* shows a similar result, and there will be a novel isoform including unannotated exon (Figure 8D and Supplementary Figure S11).

The fifth and sixth ranked genes were *NFE2L3* and *FEZ2*, respectively (Figure 8B and C). There were several reads mapped to the intron region in E3 cells and E4 cells, respectively, but spliced aligned reads were not observed between these genes exon and these intron regions. In particular, there were numerous spliced aligned reads in the intron region of *FEZ2* in E4 cells (Supplementary Figure S11), which suggest the existence of unannotated short gene.

The eighth ranked gene was *TRAM2*, and the high coverage in the intron region continued from the adjacent exon was observed mainly in E3 cells (Figure 8E). Because spliced aligned reads were not observed between the intron region and opposite exon, the coverage pattern suggests the existence of novel exon corresponds to 3′-UTR end. The similar result was also observed in the 16th ranked gene *TPX2* (Figure 8F).

## DISCUSSION

In this research, we developed a novel computational approach for differential expression analysis of scRNA-seq data based on matrix factorization of mapped count data to discover overlooked DE gene regions. Matrix factorization methods, such as principal component analysis, are a practical approach to extract essential structures and uncover biological knowledge from large-scale biological data (50). To take advantage of the large number of cells assayed in scRNA-seq data, we proposed an NMF-based approach to extract reproducible patterns and quantify differences in these patterns among groups. In particular, we used non-negative constraint to quantify DE patterns while preserving information about the group in which the patterns were expressed, and we developed a score that identifies ODEGRs by using positive maximum and negative minimum values. Such computational approaches which utilize numerical constraints based on the biological subjects can facilitate further omics studies.

We applied our algorithm to three scRNA-seq datasets and discovered several unannotated DE patterns, including DE antisense transcripts. In addition, our algorithm utilized mapping patterns in intron regions to discover overlooked alternative TSS patterns. Specifically, we detected an unannotated transcript which is a key factor for regulating differentiation. Thus, our approach has the potential to identify essential overlooked DE genes.

Although our algorithm was able to identify several intriguing ODEGRs, it remains difficult to distinguish the cause of DE transcripts such as those associated with antisense transcripts or the long unannotated transcripts of adjacent genes. In addition, the detected ODEGRs are few, and thus the impact on whole expression analyses is quantitatively small. However, our approach can discover novel transcripts and will enable further experimental and computational analyses of these transcripts, which will deepen the current understanding of the complex gene expression landscape.

As shown in the validation of alternative isoform expression, our algorithm overlooked several genes with alternative isoform expression. One limitation of our algorithm is that its detection of changes involves small exons, because small changes have little effect on the objective function and are overlooked in matrix factorization. In addition, we used the count data with a 100-bp bin size (see the ‘Materials and Methods’ section). Although our algorithm is robust to bin size (see Supplementary Paragraph 2), the differences in some small exons will be overlooked even if small bin size. This problem might be solved by using 1-bp resolution data matrix, NMF computational time and data size increase substantially with increases in matrix size, so additional improvements, such as online NMF to reduce computational time, are therefore necessary. We can also decrease the computational time by filtering out bins with low read counts, but we have to be careful so that we do not overlook DE regions in low read coverage regions like 5′ intron region of *GRB10* (Figure 7D). Moreover, our algorithm overlooks DE patterns in the filtered regions such as those with gene overlap or those with low mappability. Therefore, other approaches, such as methods based on exon–exon junction reads (18,19), will be useful to make up for each other’s weak points and to complement annotation-based analyses.

In single-cell technologies, there are two different types of technologies: one is a high-throughput scRNA-seq technology with cell barcodes and unique molecular identifiers (UMIs) that can analyze a huge number of cells, and the other is full-length scRNA-seq technology that can quantify accurate expression of each cell. In this research, we used the full-length scRNA-seq data. In comparison to the high-throughput scRNA-seq data that are usually regarded as zero-inflated data, full-length scRNA-seq data show high-quality and are not zero-inflated (see Supplementary Paragraph 3). However, it is possible to quantify the expression of each cell with shallow read depth to analyze a large number of cells, which results in a zero-inflated data matrix. In such a case, sparse NMF might be an efficient approach to analyze sparse data matrix. Also, our approach might be useful for analyzing single-cell data such as scATAC-seq data, and sparsity is an essential property in analyzing such data.

Several effective computational expression analysis methods for scRNA-seq data, such as for cell typing and for reconstructing differentiation trajectories, have been developed so far. In this research, we have proposed a novel application of scRNA-seq data for discovering overlooked DE transcripts. Here, we have developed an algorithm for differential expression analysis between two groups, and this approach might be useful for analyzing cellular heterogeneity and discovering transcripts with an overlooked multimodal distribution.

## CONCLUSION

The elucidation of hidden transcript diversity is important (12), and we have developed an algorithm to discover overlooked DE gene regions from scRNA-seq data in this study. First, we confirmed that our algorithm could detect complex DE patterns such as simulated local differential expression and alternative isoform expression. Then, we applied our algorithm to three single-cell full-length RNA-seq datasets and discovered intriguing examples of differential expression, including a transcript related to the modulation of NSPC differentiation. Our approach complements annotation-based analysis and is an effective approach for better understanding cellular regulatory mechanisms using single-cell studies.

## DATA AVAILABILITY

The sequencing data of hNSC-NC dataset can be accessed at the Gene Expression Omnibus under accession code GSE125288 https://www.ncbi.nlm.nih.gov/geo/query/acc.cgi?acc=GSE125288. The processed mES-PrE dataset and hNSC-NC dataset are available at https://doi.org/10.6084/m9.figshare.7410509.v1 and https://doi.org/10.6084/m9.figshare.7410512.v1, respectively. The software ODEGRfinder is available at GitHub https://github.com/hmatsu1226/ODEGRfinder.

## NOTES ADDED IN PROOFS

*Ethics approval and consent to participate:* The use of human iPSCs derived NSPCs was approved by ethics committees at Keio University School of Medicine (admission numbers; 20130146).

## Supplementary Material

lqz020_Supplemental_Files
