# Supplementary material for: An NMF-based approach to discover overlooked differentially expressed gene regions from single-cell RNA-seq data
Source: NAR Genom Bioinform. 2019 Dec 16;2(1):lqz020. doi: 10.1093/nargab/lqz020 (PMC8499053; doi:10.1093/nargab/lqz020)
Supplement: lqz020_Supplemental_Files [file lqz020_supplemental_files.zip › supplementary_text.pdf]

Supplemental text for "An NMF-based approach to discover overlooked differentially expressed gene regions from single-cell RNA-seq data"

Hirotaka Matsumoto Tetsutaro Hayashi Haruka Ozaki Koki Tsuyuzaki
Mana Umeda Tsuyoshi Iida Masaya Nakamura Hideyuki Okano
Itoshi Nikaido

November 22, 2019

1 hNSC-NC dataset

We performed single-cell RNA sequencing (scRNA-seq) for a neural stem cell (NSC) population derived from human induced pluripotent stem (iPS) cells. The population was heterogeneous as shown in Fig.S1. We clustered cells into subgroups and defined cell types based on the expression of marker genes, which resulted in the NSC subgroup (red), neural cell (NC) subgroup (yellow), and Niche cell subgroup (green). We also identified some uninterpretable subgroups (pink, cyan, and purple) that are thought to be experimental artifacts. In this research, we investigated differential expression between 515 NSCs and 80 NCs.

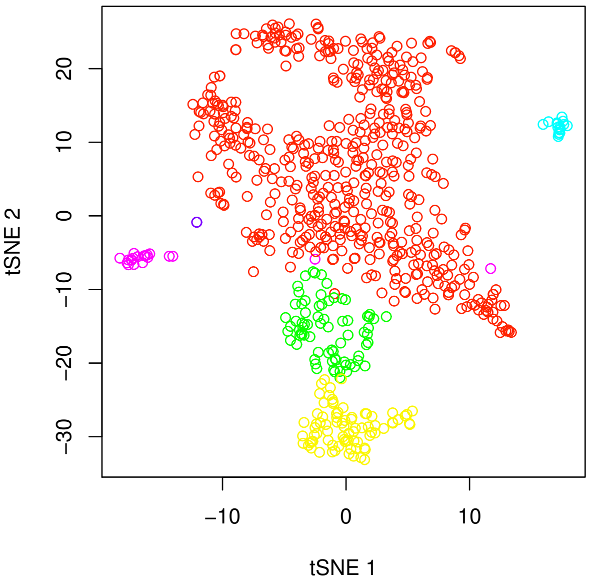

Figure S1: The t-distributed stochastic neighbor embedding analysis of the iPS cell derived neural stem cell population, colored by cluster assignment.

2 Selection of bin size

In the main text, our analysis used a read count data matrix with 100-bp bin size. In this section, we evaluate the robustness of our algorithm to bin size. We applied our algorithm to the mES-PrE count data matrices with 50-bp and 200-bp bin sizes, respectively. As a result, $\Delta T_{\mathrm{NMF-TPM}}$ with 100-bp bin size was highly correlated with results using 50-bp and 200-bp bin sizes (Pearson's correlation coefficients were approximately 0.92 and 0.91, respectively) (Fig.S2). In addition, all of the top-ranked genes based on the 100-bp bin size results (i.e., *Zmynd8*, *Utrn*, *Echdc2*, *Brd1*, *Macf1*, and *Gata6*) are ranked within the top 10 in the analyses using both 50-bp and 200-bp bin sizes. Thus, characteristic novel differentially expression regions (DERs) can be detected regardless of bin size, and our algorithm is robust to bin size.

As discussed in the "Validation on simulation dataset" section in the main text, our analysis might overlook some small DERs. Although we can detect some overlooked DERs by using a smaller bin size, the computational time for count data with a small bin size becomes large. The computational complexity of NMF at each iteration is generally $\mathcal{O}(CLK)$ (where C, L, and K are the numbers of cells, bins, and ranks, respectively), and therefore, the computational time for a data matrix with 1-bp resolution will become 100 times larger than that for a data matrix with a 100-bp bin size. The running time for the hNSC-NC dataset for 1,000 gene regions was about 10 h, and that for 1-bp resolution data would exceed a month. For the above reasons, we used data matrices with 100-bp bin size to find DERs in order to make the running time feasible.

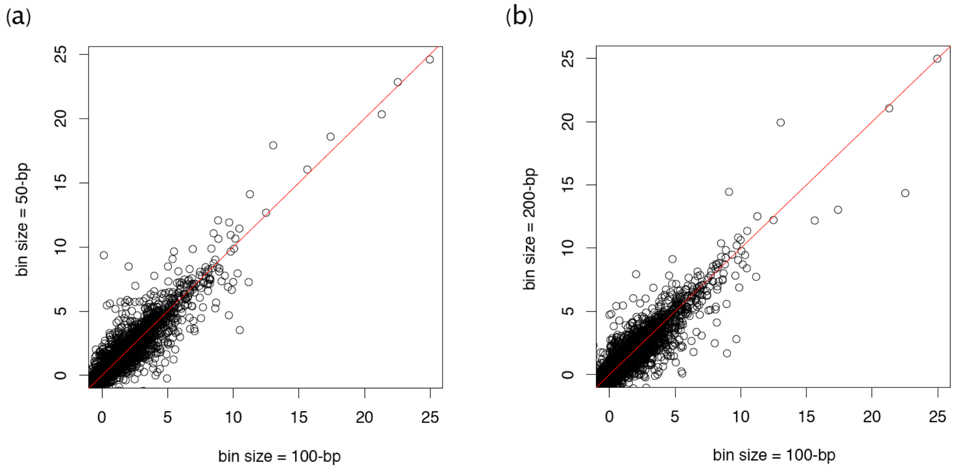

Figure S2: Comparison of $\Delta T_{\mathrm{NMF-TPM}}$ with 100-bp bin size and 50-bp bin size (a) and 100-bp bin size and 200-bp bin size (b) (We visualized $\Delta T_{\mathrm{NMF-TPM}}$ of high-ranking genes to visualize consistency between $\Delta T_{\mathrm{NMF-TPM}}$ with different bin sizes).

3 Distribution of the read counts

We plotted the distribution of read counts for gene regions to investigate the sparseness of full-length scRNA-seq data [we applied a $\log_{10}(\mathrm{count}+1)$ transformation to the read count values of a cell c for a bin b in a gene g and described it with $\mathbf{X}^{(g)}_{c,b}$]. Because we cannot distinguish missing values from actual zero values, we filtered differentially expressed or low-expressed gene regions. We filtered out any gene g whose p-value was lower than 0.01 in a t-test of the mean of the read count for the gene region ($\frac{1}{L_g}\sum_b \mathbf{X}^{(g)}_{c,b}$, where L_g is the number of bins in the gene region) between cell types. We also filtered out any gene g that satisfies $\frac{1}{CL_g}\sum_c\sum_b \mathbf{X}^{(g)}_{c,b} < 0.5$ to remove low-expressed genes. The histograms of the mean of the logarithm of the

read count ($\frac{1}{L_g}\sum_b \mathbf{X}_{c,b}^{(g)}$) for gene regions in each dataset are shown in Fig.S3(a-c). These histograms show that the values are not zero-inflated. In comparison to the high-throughput 3′-end scRNA-seq, which has zero-inflated expression data, full-length scRNA-seq will be less zero-inflated.

We also investigated the distribution of the read count for bins. In addition to the above filtering, we also filtered out any bin b that satisfies $\frac{1}{C}\sum_c \mathbf{X}_{c,b}^{(g)} < 3$ to remove low-mapped bins. The histograms of the logarithm of the read count ($\mathbf{X}_{c,b}^{(g)}$) for bins in each dataset are shown in Fig.S3(d-f). These histograms show that the number of bins with zero coverage is small, and the read count for bins with high-expressed genes are not zero-inflated. However, the number of zero values increased if we included low-mapped bins, and therefore low-mapped bins such as those corresponding to intron regions will be slightly zero-inflated.

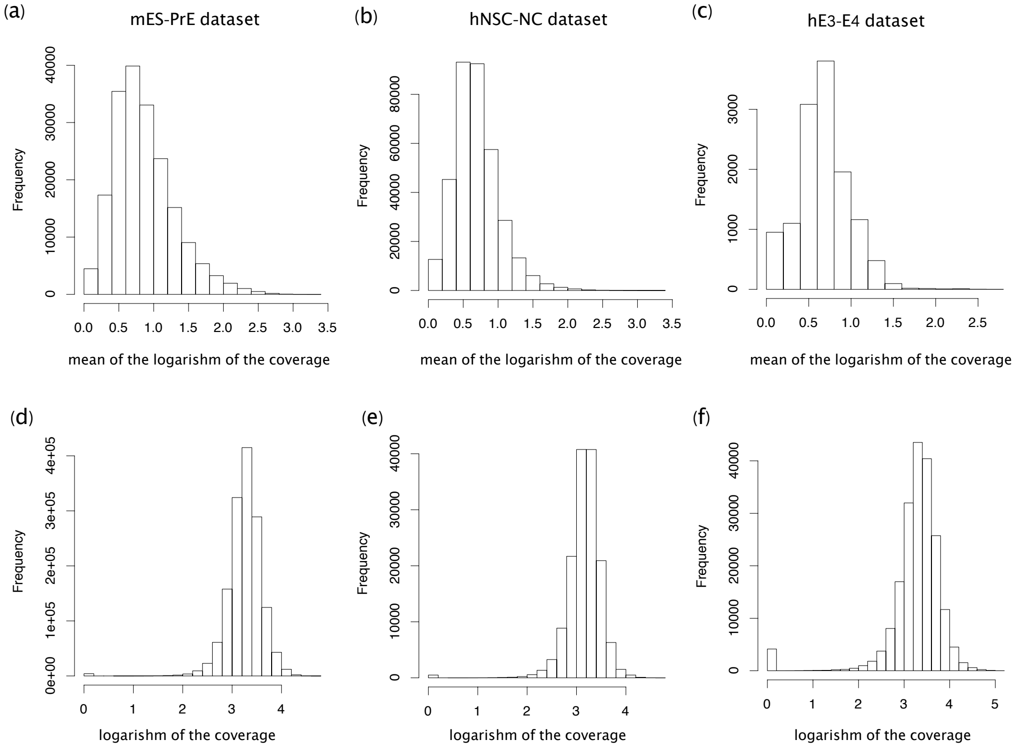

Figure S3: The histograms of the mean of the logarithm of the read count for gene regions ($\frac{1}{L_g}\sum_b \mathbf{X}_{c,b}^{(g)}$) for the mES-PrE dataset (a), the hNSC-NC dataset (b), and the hE3-E4 dataset (c), and the histograms of the logarithm of the read count for bins ($\mathbf{X}_{c,b}^{(g)}$) for the mES-PrE dataset (d), the hNSC-NC dataset (e), and the hE3-E4 dataset (f).

3.1 Distribution of the NMF coefficient matrix

We also investigated the properties of the NMF mixture coefficient matrix ($\mathbf{H}$) and plotted the histogram of $\mathbf{H}_{k,\cdot}$, which corresponds to $\Delta T_{\mathrm{NMF-TPM}}$. The histograms for the high-ranking genes in the hNSC-NC dataset, which contains the highest number of cells in the three datasets, are shown in Fig.S4. In short, the values of $\mathbf{H}_{k,c}$ that correspond to the high-expressed cell type were not zero-inflated, and therefore sparseness is not a substantial complication in quantifying differential expression based on NMF coefficients. However, the values of NSCs for *GRB10* are zero-inflated. The DER in *GRB10* is the 5′-end intron region (Fig.7(d)), and the reads in the DER are likely to be derived from a long pre-mRNA. The number of reads that correspond to the pre-mRNA is small, and the coefficients are zero-inflated for such regions with low coverage. NMF is

used for imputing missing values, and therefore, even if read counts of some bins for a particular DER are missing, the coefficient will be reconstructed based on the read counts of other bins corresponding to the DER. Additionally, we can detect DERs if the mean values of $\mathbf{H}$ are significantly different between two cell types, and our algorithm can thus find DERs like *GRB10*.

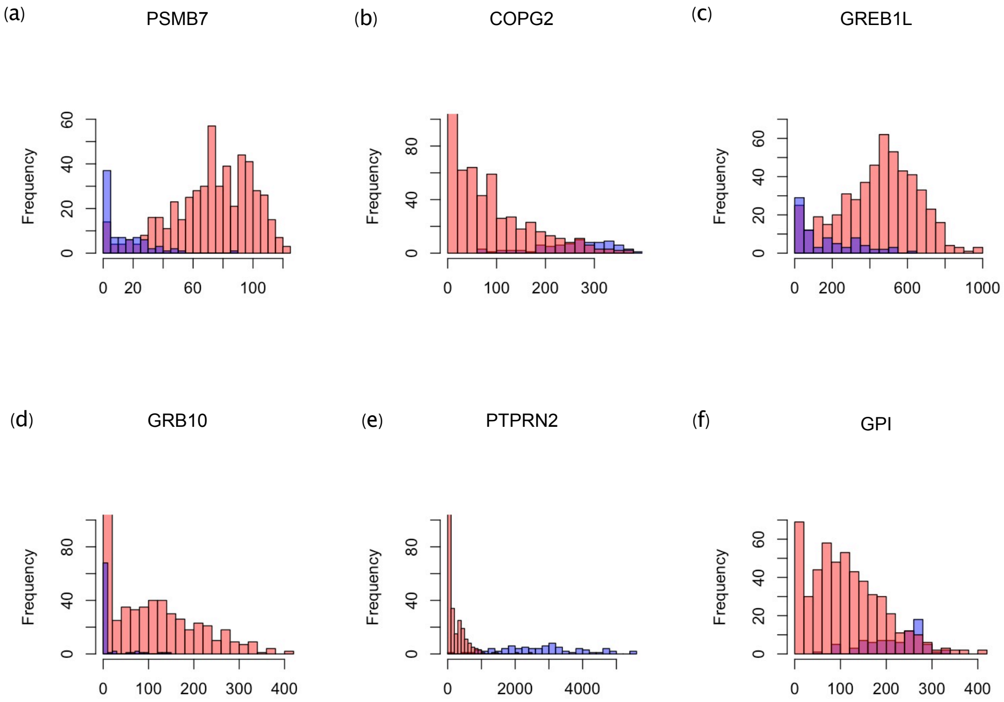

Figure S4: The histograms of the NMF coefficients ($\mathbf{H}$), which correspond to $\Delta_{\mathrm{NMF-TPM}}$. The red and blue bars represent counts for NSCs and NCs, respectively. For visibility, the maximum values of the y-axis are set to 100 for *COPG2*, *GRB10*, and *PTPRN2*.

4 Validation of T^+_{NMF} and T^-_{NMF} with a simulation dataset

We evaluated whether the maximum and minimum values of t-statistics (T^+_{NMF} and T^-_{NMF}) are reasonable values for quantifying local differential expression. To accomplish this, we used the simulation dataset in the main text, which contains the local DER of length L' in the read count matrix. We quantified the level of genuine differential expression based on the difference in the mean read count for the local DER (T_{Mean}). T^+_{NMF} (or T^-_{NMF}) is generally consistent with the T_{Mean} for the DER (the correlation coefficients are 0.73, 0.77, and 0.77 for the simulation dataset with $L' = 100$, 50, and 10, respectively) (Fig.S5). Because T^+_{NMF} and T^-_{NMF} are almost equivalent to the T_{Mean} of the DER for most of the data, these values that quantify the differences based on the NMF coefficient matrix will be reasonable indicators for quantifying the differential expression of local DERs.

However, about 2% of the count data (25, 27, and 18 data points from the simulation dataset with $L' =$ 100, 50, and 10, respectively) showed that T^+_{NMF} (or T^-_{NMF}) was more than twice as large as T_{Mean}. It is notable that about half of such overestimated values were derived from the coefficient matrices that correspond to $K = 2$ or $K = 5$. Therefore, these separations are suggested not to be caused by over-decomposing the read count matrices (i.e., setting large K) but by the denoising of count data, which introduce large T^+_{NMF} (or

T^-_{NMF}) values in comparison to the T values based on the mean count data. Although this might not represent a case of overfitting, our purpose is to detect overlooked DERs, and we thus filtered out large ΔT values if a particular DER is sufficiently detected by reference-based DE analysis (see Algorithm 1 in "Discovery of ODEGRs" section).

We also evaluated the distribution of T^+_{NMF} and T^-_{NMF} for label-shuffled data to investigate whether these values become large by chance (we used $L' = 100$ data). We calculated T^+_{NMF} and T^-_{NMF} values based on the shuffled label set, and Fig.S5 (d) shows the comparison of the values for the original label and the shuffled label. All of the values for the shuffled label were below those of the original label, and therefore it is unlikely that the values become large by chance owing to over-decomposing of the read count matrix. Thus, our approach based on T^+_{NMF} (or T^-_{NMF}) can quantify the differential expression of the local DERs.

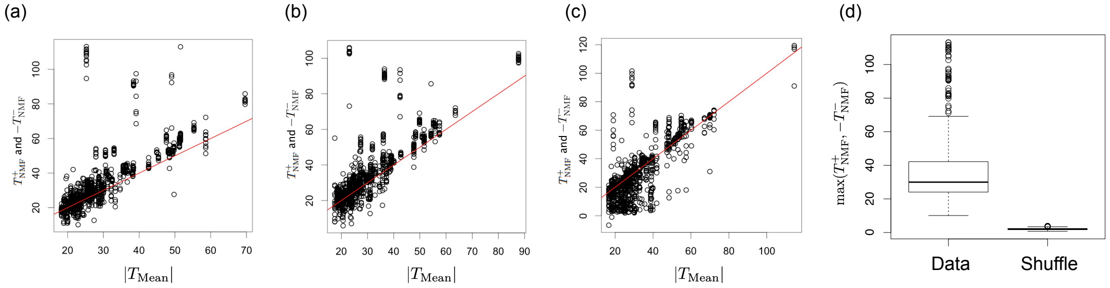

Figure S5: Comparison of t-statistics based on the mean data of the DER (T_{Mean}) and T^+_{NMF} (or $-T^-_{\mathrm{NMF}}$). (a), (b), and (c) show the results of $L' = 100$, $L' = 50$, and $L' = 10$ datasets, respectively. (d) shows the boxplot of T^+_{NMF} (or $-T^-_{\mathrm{NMF}}$) for the original data and that for the label-shuffled data.

5 Analysis of NMF- and TPM-based scores

We compared the NMF-based DE score $T^{+,-}_{\mathrm{NMF}}$ and the TPM based-DE score $T^{+,-}_{\mathrm{TPM}}$ for each dataset (Fig.S6(a),(b),(c)). In brief, $T^{+,-}_{\mathrm{NMF}}$ and $T^{+,-}_{\mathrm{TPM}}$ are consistent and strongly correlated (Pearson's correlation coefficients between the two values were about 0.83, 0.84, and 0.77, respectively), but a small number of genes had significantly different scores. When the absolute values T^+_{NMF} (or T^-_{NMF}) and T^+_{TPM} (or T^-_{TPM}) are large, the difference $T^+_{\mathrm{NMF}} - T^+_{\mathrm{TPM}}$ (or $-T^-_{\mathrm{NMF}} + T^-_{\mathrm{TPM}}$) tends to be large in several genes, which results in large $\Delta T_{\mathrm{NMF-TPM}}$ values. However, such genes are regarded as DE genes according to both scores, and their examination is contrary to our purpose of discovering overlooked DE gene regions. Therefore, we did not evaluate such differential expression in the course of describing the discovering ODEGR in the Method section of the main text.

The values of $\Delta T_{\mathrm{NMF-TPM}}$ in descending order for each dataset are shown in Fig.S6)(d),(e),(f). A small fraction of genes show large positive and negative values of $\Delta T_{\mathrm{NMF-TPM}}$, and the ODEGRs are thought to be included in the genes with large positive $\Delta T_{\mathrm{NMF-TPM}}$ values.

Our NMF-based approach tends to overlook exons with small changes owing to their small effect in the objective function and cannot detect differential expression in the filtered regions as mentioned in the main text, which results in large negative $\Delta T_{\mathrm{NMF-TPM}}$ in some genes.

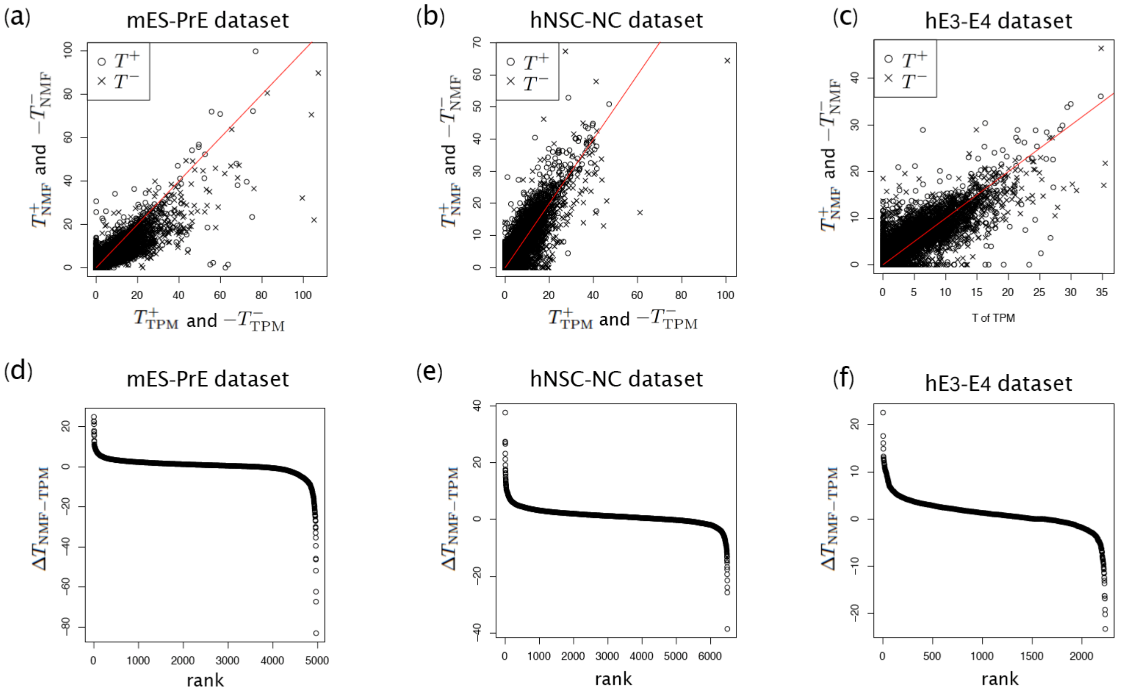

Figure S6: Comparison of T^{+}_{TPM} (or T^{-}_{TPM}) and T^{+}_{NMF} (or T^{-}_{NMF}) in (a) the mES-PrE dataset, (b) the hNSC-NC dataset, and (c) the hE3-E4 dataset. $\Delta T_{\mathrm{NMF-TPM}}$ values in rank order in (d) the mES-PrE dataset, (e) the hNSC-NC dataset, and (f) the E3-E4 dataset.

6 ODEGRfinder with sparse NMF

Sparse NMF is an effectual approach to decomposing a sparse data matrix, and we evaluated whether sparse NMF is effective in the context of our research. We used the method "snmf/l" and "snmf/r" in the NMF package, which enforce sparseness on $\mathbf{W}$ and $\mathbf{H}$, respectively.

6.1 Validation with the simulation dataset

We evaluated our algorithm based on the sparse NMF method ("snmf/l" and "snmf/r") with the simulation dataset in the main text (Fig.S7). The AUC values of our algorithm with "snmf/l" for L' =100, 50, and 10 are 0.97, 0.98, and 0.95, and those with "snmf/r" are 0.99, 0.99, and 0.93, respectively. These AUC values are almost equivalent to those generated by our algorithm with general NMF (method="lee"). However, the AUC values of the sparse NMF method for $L' = 100$ with large K are better than the AUC value of the general NMF method. Although the total performance is almost equivalent in this dataset, sparse NMF might be an effective approach in some cases.

6.2 Validation with the mES-PrE dataset

We also calculated $\Delta T_{\mathrm{NMF-TPM}}$ with "snmf/l" and "snmf/r" for the mES-PrE dataset and investigated the high-ranking gene list (we refer to $\Delta T_{\mathrm{NMF-TPM}}$ based on "lee", "snmf/l", and "snmf/r" methods as ΔT_{lee}, $\Delta T_{\mathrm{snmf/l}}$, and $\Delta T_{\mathrm{snmf/r}}$, hereafter). The correlation coefficient between ΔT_{lee} and $\Delta T_{\mathrm{snmf/l}}$ is 0.69, while that between ΔT_{lee} and $\Delta T_{\mathrm{snmf/r}}$ is 0.81. Moreover, 8 and 9 of the top 10 high-ranking genes for ΔT_{lee} were ranked in the top 30 for $\Delta T_{\mathrm{snmf/l}}$ and $\Delta T_{\mathrm{snmf/r}}$, respectively, while 9 and 8 of the top 10 high-ranking genes

for $\Delta T_{\mathrm{snmf/l}}$ and $\Delta T_{\mathrm{snmf/r}}$ were ranked in the top 30 for ΔT_{lee}. As such, most of the high-ranking genes were consistent among ΔT_{lee}, $\Delta T_{\mathrm{snmf/l}}$, and $\Delta T_{\mathrm{snmf/r}}$ results.

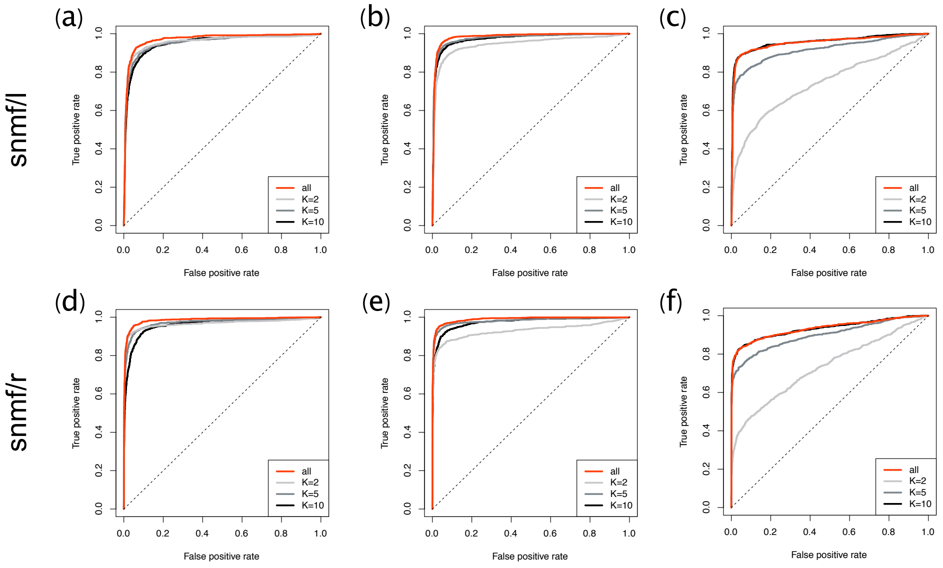

Figure S7: The ROC curves for the simulation dataset. (a-c) are the results based on the "snmf/l" method, and (d-f) are the results based on the "snmf/r" method. Simulation results for (a,d) $L' = 100$, (b,e) $L' = 50$, and (c,f) $L' = 10$, where L' is the length of local differential expression patterns.

7 $\Delta T_{\mathrm{NMF-TPM}}$ based on Salmon

In the main text, we used Sailfish for annotation-based expression quantification. We also used Salmon, which is the successor of Sailfish, to quantify the TPM matrix and compared $\Delta T_{\mathrm{NMF-TPM}}$ based on Salmon ($\Delta T^{\mathrm{Salmon}}_{\mathrm{NMF-TPM}}$) with that using Sailfish ($\Delta T^{\mathrm{Sailfish}}_{\mathrm{NMF-TPM}}$). For the hE3-E4 dataset, $\Delta T^{\mathrm{Salmon}}_{\mathrm{NMF-TPM}}$ and $\Delta T^{\mathrm{Sailfish}}_{\mathrm{NMF-TPM}}$ are highly correlated (the correlation coefficient is approximately 0.96). Moreover, all of the top 10 high-ranking genes with $\Delta T^{\mathrm{Sailfish}}_{\mathrm{NMF-TPM}}$ were ranked in top 15 with $\Delta T^{\mathrm{Salmon}}_{\mathrm{NMF-TPM}}$ and vice versa. Therefore, differences between Sailfish and Salmon could only have had a small effect on our analysis.

8 Permutation-based test for $\Delta T_{\mathrm{NMF-TPM}}$

Our DE score ($\Delta T_{\mathrm{NMF-TPM}}$) is based on the difference between t-statistics, which is an ad-hoc indicator of ODEGRs. Although we cannot derive the significance of the score theoretically, we can evaluate the significance using several approaches.

First, we can quantify the significance of the difference in the NMF coefficients between cell labels based on the t-test. Accordingly, we used the p-value of the t-test, which corresponds to $\Delta T_{\mathrm{NMF-TPM}}$ (i.e., T^{+}_{NMF} or T^{-}_{NMF}), to quantify the significance of the potential DER. Second, we can quantify the significance of $\Delta T_{\mathrm{NMF-TPM}}$ using a permutation-based approach. We shuffled the rows of the matrix, which correspond to cells, for each column (i.e., bin). Then, we conducted NMF and calculated $\Delta T_{\mathrm{NMF-TPM}}$ from the shuffled data. We regarded the distribution of $\Delta T_{\mathrm{NMF-TPM}}$ from the shuffled data as a null distribution, approximated the resulting distribution with a normal distribution, and calculated the p-value of the real $\Delta T_{\mathrm{NMF-TPM}}$ with a one-sided test. However, the above permutation-based test requires the computational time associated with NMF calculation, and therefore we also propose an alternative permutation-based test. We calculated

t-statistics and $\Delta T_{\mathrm{NMF-TPM}}$ from the NMF coefficient matrix of real data while shuffling cell labels, regarded the $\Delta T_{\mathrm{NMF-TPM}}$ distribution as the null distribution, and calculated the p-value as before. The results of each test are shown in Tab.S1.

As stated above, our DE score is an ad-hoc indicator, but we can evaluate the significance of detected DERs with such a permutation-based test and our algorithm has the potential to advance our understanding of transcript structures.

Table S1: The $-\log_{10}(p\text{-value})$ of high-ranking genes in each dataset. The first row represents t-test p-values for T^{+}_{NMF} or T^{-}_{NMF}, the second row represents the results of the permutation-test based on data shuffling, and the third row represents the result of permutation-test based on label shuffling.

	Zmynd8	*Utrn*	*Echdc2*	*Brd1*	*Macf1*	*Gata6*
t-test	70.9	44.2	62.7	47.4	57.3	23.8
data shuffled	76.4	62.0	55.4	36.8	29.6	20.6
label shuffled	55.4	45.1	40.4	27.0	21.9	15.3

	PSMB7	*COPG2*	*GREB1L*	*GRB10*	*PTPRN2*	*GPI*
t-test	61.6	33.5	24.3	50.8	31.2	22.4
data shuffled	28.3	12.8	10.2	8.0	7.9	7.5
label shuffled	27.6	12.6	10.1	8.0	7.8	7.5

	CDA	*NFE2L3*	*FEZ2*	*CCDC12*	*TRAM2*	*TPX2*
t-test	32.8	23.0	38.4	25.3	20.3	18.5
data shuffled	8.6	7.8	7.8	7.6	7.4	6.3
label shuffled	9.2	8.4	8.3	8.1	8.0	6.7

9 Discovery of ODEGRs

We investigated the ODEGRs based on their ranked $\Delta T_{\mathrm{NMF-TPM}}$ values in descending order. Even if $\Delta T_{\mathrm{NMF-TPM}}$ is large, the annotation-based approach also detects the DE when T_{TPM} is sufficiently large. Therefore, we used $\min(0, T^{+}_{\mathrm{NMF}} - T^{+}_{\mathrm{TPM}})$ instead of $T^{+}_{\mathrm{NMF}} - T^{+}_{\mathrm{TPM}}$ if $T^{+}_{\mathrm{TPM}} > 10$ and $\min(0, -(T^{-}_{\mathrm{NMF}} - T^{-}_{\mathrm{TPM}}))$ instead of $-(T^{-}_{\mathrm{NMF}} - T^{-}_{\mathrm{TPM}})$ if $T^{-}_{\mathrm{TPM}} < -10$ for calculating $\Delta T_{\mathrm{NMF-TPM}}$ to discover overlooked DE gene regions (see Algorithm 1).

Algorithm 1 Calculate $\Delta T_{\mathrm{NMF-TPM}}$

```
ΔT ← −∞
if T+_TPM > 10 then
    ΔT ← max(ΔT, min(0, T+_NMF − T+_TPM))
else
    ΔT ← max(ΔT, T+_NMF − T+_TPM)
end if
if T−_TPM < −10 then
    ΔT ← max(ΔT, min(0, −(T−_NMF − T−_TPM)))
else
    ΔT ← max(ΔT, −(T−_NMF − T−_TPM))
end if
return ΔT
```

We also considered the reproducibility of NMF results. As there is no global optimization algorithm for NMF, the result depends on the initialization. Accordingly, we calculated $\Delta T_{\mathrm{NMF-TPM}}$ for a gene with three initial values generated by different random seeds, and we used only the minimum value of $\Delta T_{\mathrm{NMF-TPM}}$ among the three trials to filter unreliable differences.

9.1 Reproducibility of NMF-based scores and examples of false-positive cases

There is no global minimization algorithm for NMF, and the results of NMF vary depend on the initialization. To detect reliable ODEGRs, we calculated $\Delta T_{\mathrm{NMF-TPM}}$ three times (described as trials "A", "B", and "C") with different random seeds. The overlap of the top 20 ranking genes are visualized with Venn diagrams (Fig.S8(a)). Over half of the genes were common among the three trials, and there were several intriguing patterns, as mentioned in the main text. However, there were some non-overlapping genes, and we investigated those that were detected in only trial "A" for the mES-PrE dataset (Fig.S8(b)(c)). In trial "A", $T^{+}_{\mathrm{TPM}} = 0.0$ and $T^{+}_{\mathrm{NMF}} = 13.9$ for *Gfpt1*, and $T^{-}_{\mathrm{TPM}} = 0.0$ and $T^{-}_{\mathrm{NMF}} = -13.4$ for *Hspa4*. Although the NMF-based scores are high, there are no clear directional DE patterns expected from the NMF-based scores, and these two genes from trial "A" are suggested to be false-positive cases. This result happens when the NMF separates the coverage data excessively, and insignificant differences are emphasized. Such false-positive cases are usually filtered using the minimum value from multiple trials.

In the hNSC-NC dataset, most of the highly ranked genes were common to all three trials. However, there are some false-positive cases as mentioned above for the common genes. The examples of such false-positive genes are *PDZD4* and *MAPRE2*, which are ranked 3rd and 7th, respectively. These cases cannot completely be filtered even if we had used the minimum value of $\Delta T_{\mathrm{NMF-TPM}}$ from multiple trials, and further improvements are necessary to reduce such false-positive results.

9.2 Examples of overlooked DE patterns included in the current annotation

As mentioned in the main text, we used the GENCODE vM9 annotation, as described in our previous work, to analyze the mES-PrE dataset. The overlooked DE transcripts based on vM9 might be included in the current transcript annotations. Therefore, we reanalyzed the annotation-based approach with the current GENCODE vM18 annotation. Thus, the DE transcripts of some ODEGRs based on vM9 were detected using the current vM18 annotation. The first example is *Utrn*, which ranked 2nd, and the $T^{+,-}_{\mathrm{TPM}}$ values based on the vM18 annotation were 46.2 and -15.5, which were significantly higher and lower, respectively, than those based on the vM9 annotation ($T^{+,-}_{\mathrm{TPM}}$ values for vM9 were 0.0 and -4.4, respectively) (Fig.S9(a),(b)). The second example is *Arid5b*, which ranked 7th, and the $T^{+,-}_{\mathrm{TPM}}$ values based on the vM18 annotation were 13.2 and -11.5, which were significantly higher and lower, respectively, than those based on the vM9 annotation ($T^{+,-}_{\mathrm{TPM}}$ values for vM9 were 1.1 and -0.0) (Fig.S9(c),(d)). The third example is *Macf1*, which ranked 5th and corresponded to a region to which numerous PrE cell reads were mapped, and the region is annotated as exon in only the vM18 annotation (Fig.S9(e),(f)).

These results show the validity of the high-ranking results and the potential of our algorithm to discover unannotated alternative splicing events.

9.3 Examples of overlooked DE patterns from unannotated long transcripts of adjacent genes

Some ODEGRs corresponding to high-ranking genes suggest the existence of previously unannotated long transcripts of adjacent genes, as mentioned in the main text. The key example in the mES-PrE dataset is *Echdc2*, which ranked 3rd, and the key example in the hNSC-NC dataset is *PSMB7*, which ranked 2nd. The mapping count data and annotation are visualized, including their adjacent gene regions, in Fig.S10. For both genes, several reads were mapped to the 3′-end intron regions, to which only transcripts of the example genes correspond. Therefore, the reads are apparently derived from transcripts containing the retained intron of the genes. However, the mapped coverage continued from the adjacent genes, and the coverage of the 3′-end intron regions are correlated with the coverage of adjacent genes. Therefore, such reads are suggested to be

derived from unannotated long transcripts of adjacent genes. However, there is also the possibility that these reads correspond to other novel transcripts, and further experimental validation is necessary to reveal the complete structure of these unannotated transcripts.

Such unannotated long transcripts have potential roles in gene regulation and tissue-specific imprinting, as occurs with *Copg2* and the long transcript of *MestXL* as mentioned in the main text. Thus, the detection of such phenomena is an important subject in differential expression analysis.

9.4 Examples of split aligned reads in ODEGRs

We found several high coverage regions in the intron regions of the highly ranked ODEGRs, especially in the hE3-E4 dataset. These patterns suggest the existence of unannotated exons. If these were unannotated exons, there would be numerous split aligned reads between these intron regions and annotated exons of these genes. Therefore, we visualized the coverage and split aligned reads with Sashimi plot in some ODEGRs in the hE3-E4 dataset (Fig.S11). As mentioned in the main text, there were numerous split aligned reads between the intron regions and exons in *CDA* and *CCDC12*. In contrast, such split aligned reads were not observed in *NEF2L3* and *FEZ2*. In particular, there were several split aligned reads inside the intron region in *FEZ2*.

9.5 Validation of unannotated transcripts with qRT-PCR

We confirmed cell type-specific expression of unannotated transcripts discovered in our analysis using qRT-PCR. We investigated three unannotated transcripts in the *Zmynd8*, *Brd1*, and *Echdc2* regions for the mES-PrE dataset. Mouse ES cells and PrE cells were cultured according to the previously reported [1]. RNA extraction was performed by using a Direct-zol RNA Miniprep kit (Zymo Research) from three different culture dishes in each condition. Reverse transcription was performed using a SuperScript IV VILO Master Mix with ezDNase Enzyme (Thermo), using 100 ng of total RNA per reaction; each condition was set up in triplicate as biological replicates. Real time PCR was performed using a QuantiTect SYBR Green PCR Kit (Qiagen) on a Light Cycler 480 (Roche) according to the instructions. qPCR primers were designed using the Universal ProbeLibrary Assay Design Center. In *Zmynd8* and *Brd1* assay, we used primer sets according to previously reported [2]. All primer sets are listed in the supplementary data. Relative quantification of transcript expression was carried out by the $2^{-\Delta\Delta \mathrm{CT}}$ method.

We quantified the expression of the unannotated transcripts in *Zmynd8* and *Brd1* regions using primer sets for the ES cell-specific highly mapped intron regions. We also quantified the expression of *Zmynd8* and *Brd1* using primer sets for the exons correspond to the short, long, and both isoforms. As a result, we confirmed the ES cell-specific expression of the unannotated transcripts (Fig.S12 (a,b)).

To validate the unannotated transcript in the *Echdc2* region, we used 24 primer sets designed for the region, range from *Echdc2* $3'$ intron region to *Zyg11a* gene region (Fig.S12(c)). As a result, we confirmed the ES cell-specific expression of the primer sets, not corresponding to exons of *Echdc2* and *Zyg11a*, which support the existence of the unannotated transcript in the region (Fig.S12(d)). In addition, we investigated the direction of reads corresponding to the unannotated transcript using bulk rRNA-depleted total RNA-seq (rdRNA-seq) for ES sample [1] and confirmed that these reads were mapped to reverse strand that corresponds to the direction of *Zyg11a* (Fig.S13(a)). Although some regions showed low coverage, such as the region immediately downstream of *Zyg11a*, the expression of the region was confirmed by qRT-PCR (primer set id 21) (Fig.S13(b) and Fig.S12(d)). Therefore, an unannotated long isoform of *Zyg11a*, which overlaps with the *Echdc2* region, is suggested to exist. However, there is a possibility that this unannotated transcript does not connect to *Zyg11a* isoform, and it corresponds to a different novel transcript, and further experimental validation, such as long-read direct RNA sequencing, is necessary to determine the complete structure.

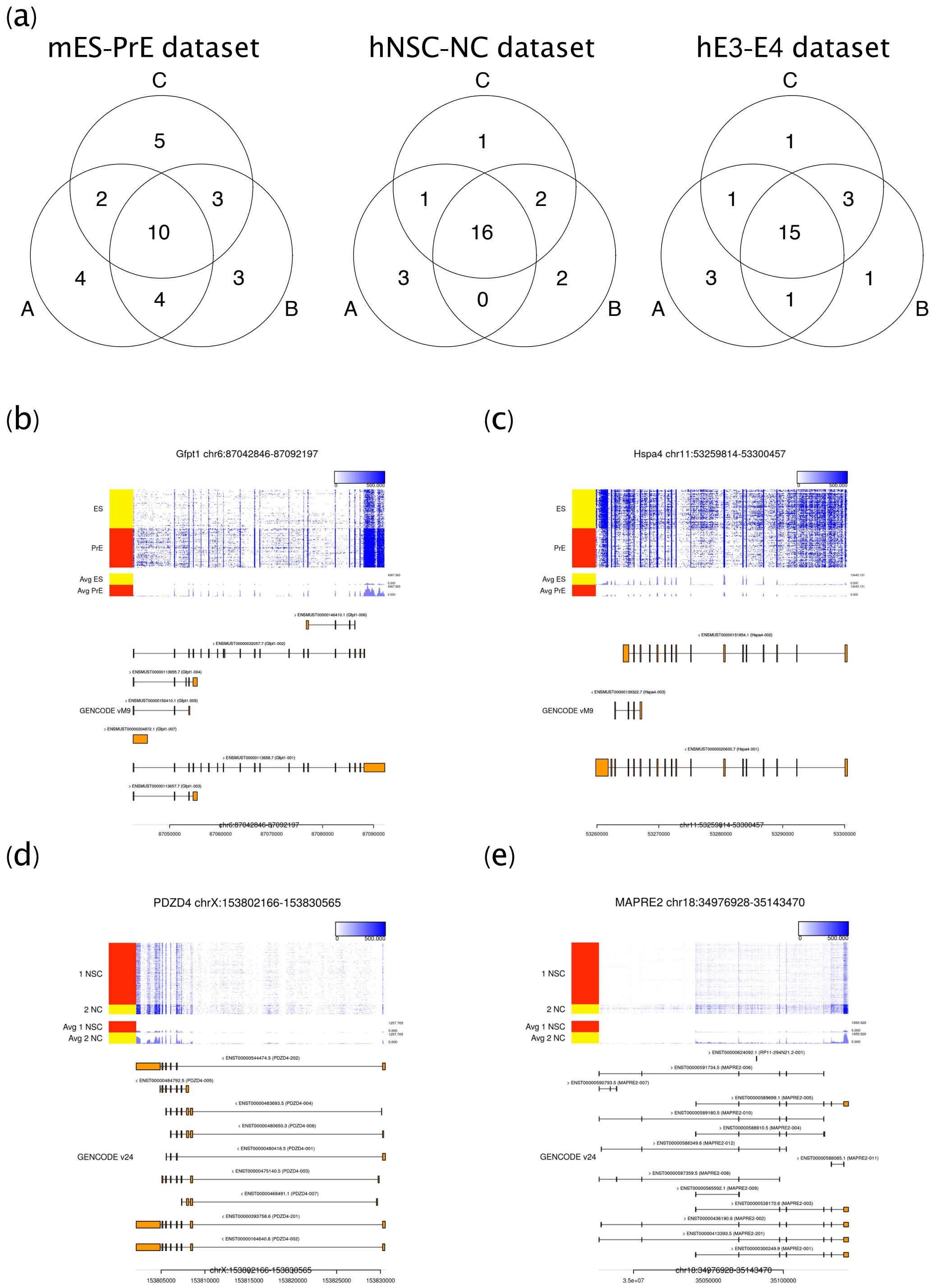

Figure S8: (a) Venn diagrams of the top 20 ranked genes by $\Delta T_{\mathrm{NMF-TPM}}$ values in three trials with different initialization seeds for the mES-PrE dataset, the hNSC-NC dataset, and the hE3-E4 dataset. (b) and (c) Examples of genes detected in only trial "A" in the mES-PrE dataset. (d) and (e) False-positive examples in the hNSC-NC dataset.

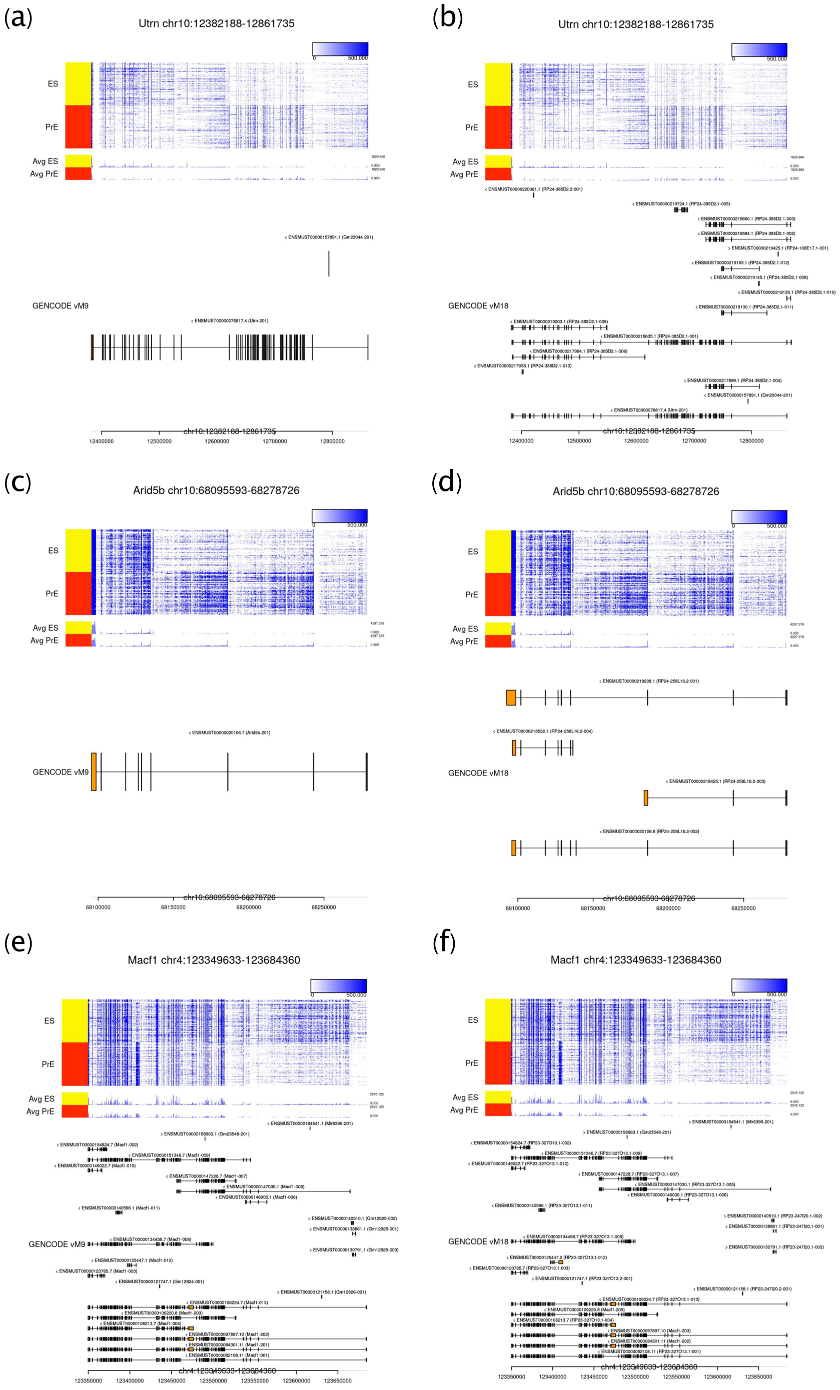

Figure S9: Examples of unexpected patterns included in the current annotation. (a) and (b) are the result of *Utrn*; (c) and (d) are the result of *Arid5b*. The left-side plots (a) and (c) correspond to the GENCODE vM9 annotation, and the right-side plots (b) and (d) correspond to the GENCODE vM18 annotation.

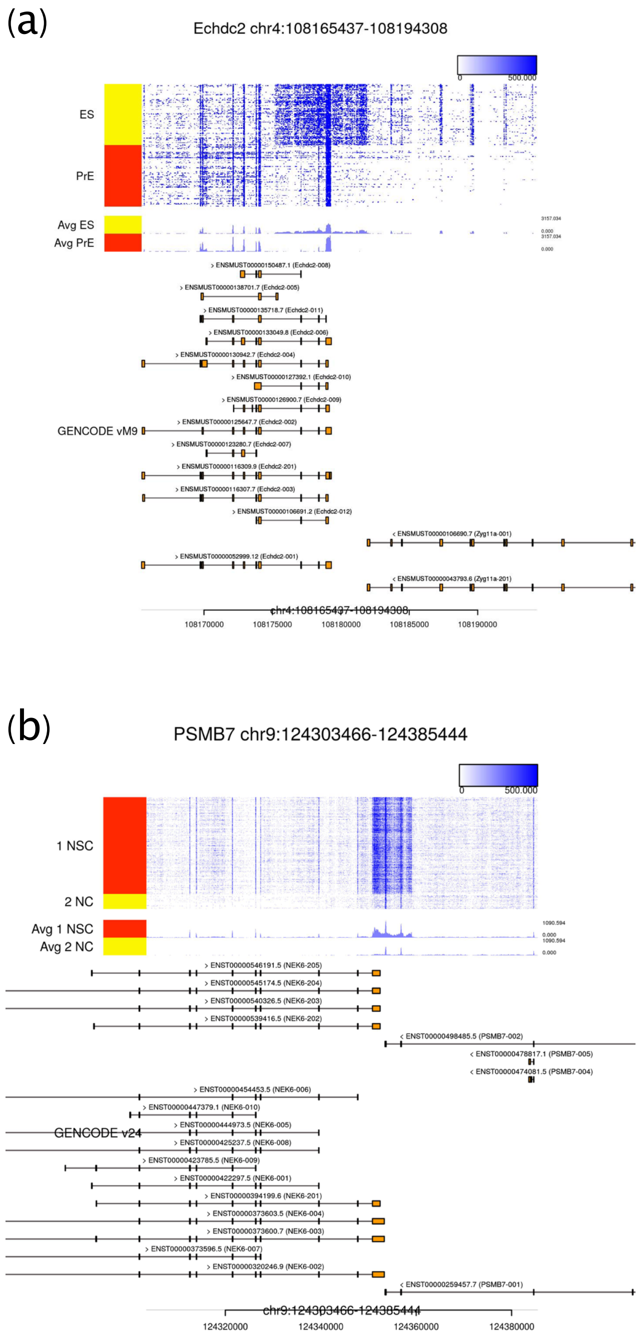

Figure S10: Examples of unexpected patterns derived from unannotated long isoforms of adjacent genes. (a) is the result of *Echdc2* in the mES-PrE dataset, and (b) is the result of *PSMB7* in the hNSC-NC dataset. We extended the visualized regions so that mapping patterns of adjacent genes are included.

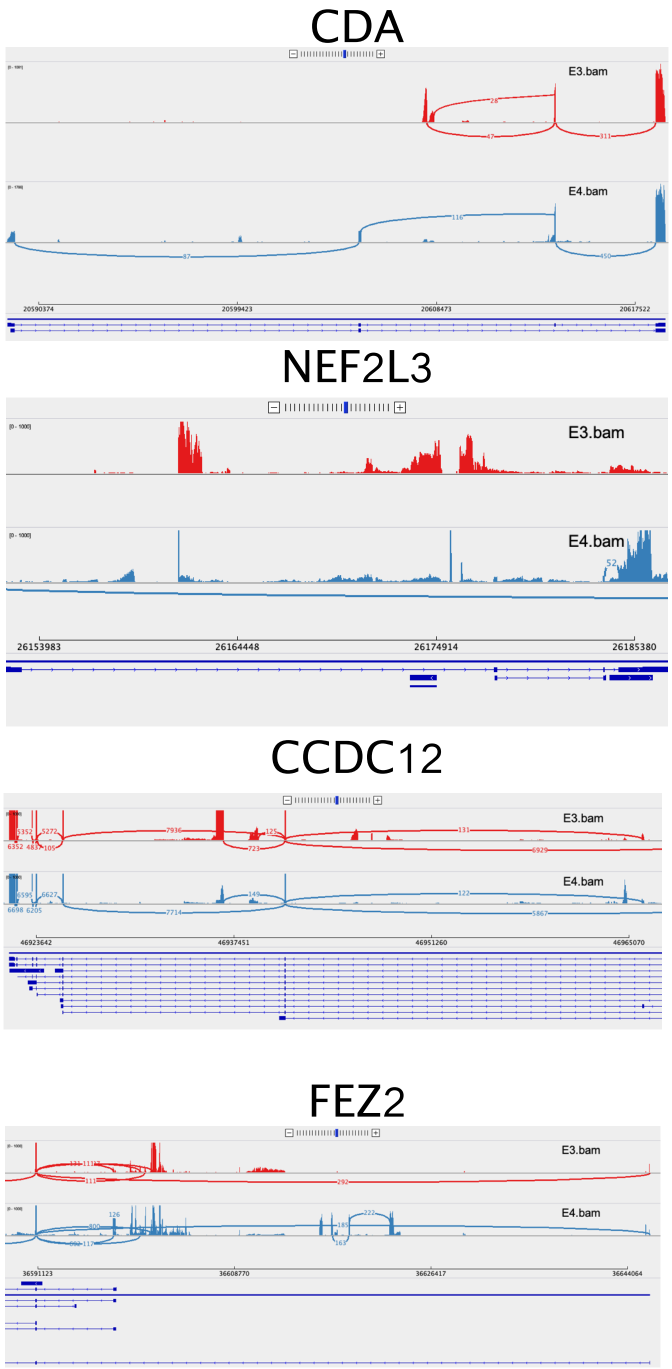

Figure S11: Sashimi Plot for highly ranked genes (*CDA*, *NEF2L3*, *CCDC12*, and *FEZ2*) in hE3-E4 dataset to visualize read coverage and split aligned reads. The mapping data of 80 E3 cells and 190 E4 cells are merged, respectively. The red and blue results correspond to E3 cells and E4 cells, respectively.

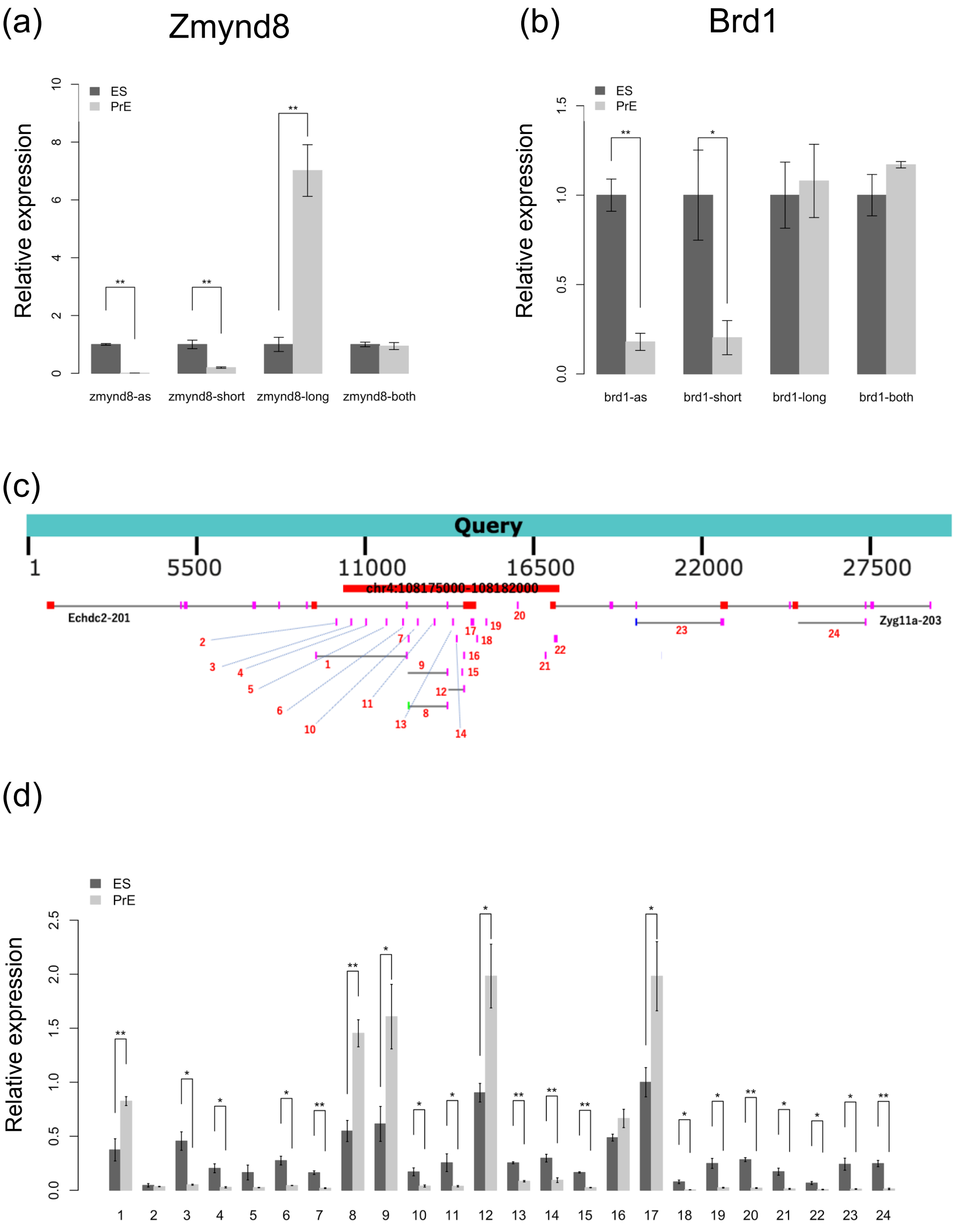

Figure S12: The relative expression of each primer sets of (a) *Zmynd8* and (b) *Brd1* region for ES and PrE cells. The relative expression were quantified with the $2^{-\Delta\Delta \mathrm{CT}}$ method. We used the value of *Gapdh* for normalization and then used the mean value of ES cell for deriving relative expression for each primer sets. The primer sets zmynd8-as and brd1-as correspond to the ES cell-specific highly mapped intron region, and other primer sets correspond to the exon in short, long, and both isoforms of each gene [2]. (c) All primer sets used for validation of the unannotated transcript in *Echdc2* 3′ region. (d) The relative expression of the above primer sets for ES and PrE cells. We used the mean value of ES cells of primer set correspond to *Echdc2* exon (primer id 17) to derive relative expression.

(a)

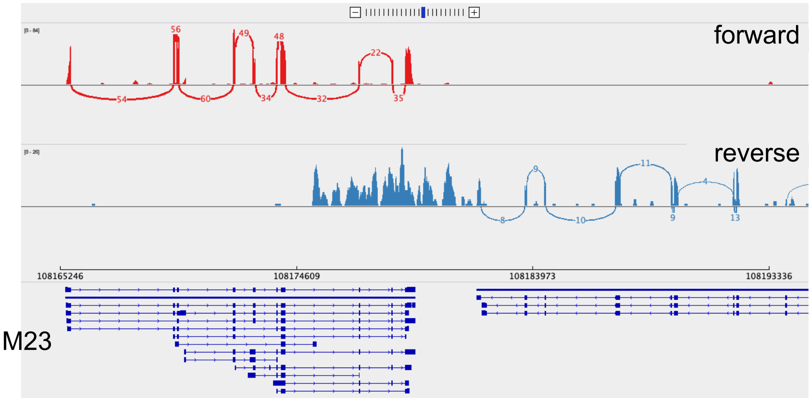

(b)

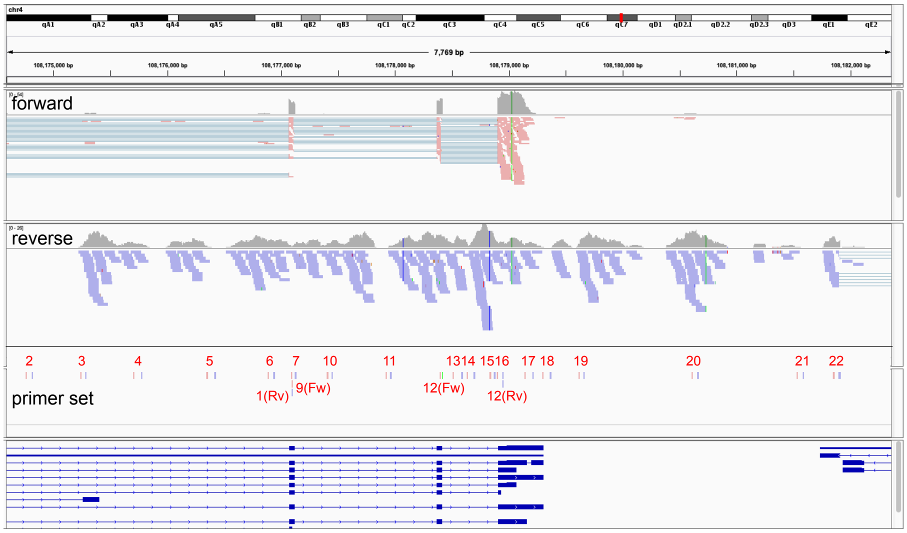

Figure S13: (a) The read coverage and the number of split-aligned reads for forward strand (red) and reverse strand (blue) of bulk rdRNA-seq for ES cells is visualized with Sashimi Plot. (b) The read coverage and position of primer sets for qRT-PCR are visualized with Integrative Genomics Viewer.

References

[1] Hayashi, T., Ozaki, H., Sasagawa, Y., Umeda, M., Danno, H., and Nikaido, I. (02, 2018) Single-cell full-length total RNA sequencing uncovers dynamics of recursive splicing and enhancer RNAs. *Nat Commun,* **9**(1), 619.

[2] Onodera, C. S., Underwood, J. G., Katzman, S., Jacobs, F., Greenberg, D., Salama, S. R., and Haussler, D. (2012) Gene isoform specificity through enhancer-associated antisense transcription. *PLoS ONE,* **7**(8), e43511.
